# Rational design of NIR-II molecule-engineered nanoplatform for preoperative downstaging and imaging-guided surgery of orthotopic hepatic tumor

**DOI:** 10.1186/s12951-023-02263-w

**Published:** 2023-12-18

**Authors:** Qi Pan, Ke Li, Xueqin Kang, Kaixuan Li, Zihe Cheng, Yafei Wang, Yuye Xu, Lei Li, Na Li, Guilong Wu, Sha Yang, Shuo Qi, Guodong Chen, Xiaofeng Tan, Yonghua Zhan, Li Tang, Wenhua Zhan, Qinglai Yang

**Affiliations:** 1 Center for Molecular lmaging Probe, Hunan Province Key Laboratory of Cancer Cellular and Molecular Pathology, Hengyang Medical School, Cancer Research lnstitute, University of South China, Hengyang, 421001 China; 2 Medical Imaging Department, The Second Affiliated Hospital of Xi’an Medical University, Xi’an, 710038 China; 3 Xi’an Key Laboratory for Prevention and Treatment of Common Aging Diseases, Translational and Research Centre for Prevention and Therapy of Chronic Disease, Institute of Basic and Translational Medicine, Xi’an Medical University, Xi’an, 710021 China; 4 School of Life Science and Technology, Engineering Research Center of Molecular & Neuro Imaging of the Ministry of Education, Xidian University, Xi’an, 710126 China; 5 Radiology Department, Ninth Affiliated Hospital of Medical College of Xi’an Jiaotong University, Xi’an, 710054 China; 6 Department of Hepatopancreatobiliary Surgery, Hengyang Medical School, The First Affiliated Hospital, University of South China, Hengyang, 421001 Hunan China; 7 Key Laboratory of Tropical Medicinal Plant Chemistry of Ministry of Education, College of Chemistry and Chemical Engineering, Hainan Normal University, Haikou, 571158 China; 8 Department of Radiation Oncology, General Hospital of Ningxia Medical University, Yinchuan, 750004 China

**Keywords:** Hepatic tumor, Imaging guidance, Photothermal therapy, Downstaging, Surgical resection

## Abstract

Orthotopic advanced hepatic tumor resection without precise location and preoperative downstaging may cause clinical postoperative recurrence and metastasis. Early accurate monitoring and tumor size reduction based on the multifunctional diagnostic-therapeutic integration platform could improve real-time imaging-guided resection efficacy. Here, a Near-Infrared II/Photoacoustic Imaging/Magnetic Resonance Imaging (NIR-II/PAI/MRI) organic nanoplatform IRFEP-FA-DOTA-Gd (IFDG) is developed for integrated diagnosis and treatment of orthotopic hepatic tumor. The IFDG is designed rationally based on the core “S-D-A-D-S” NIR-II probe IRFEP modified with folic acid (FA) for active tumor targeting and Gd-DOTA agent for MR imaging. The IFDG exhibits several advantages, including efficient tumor tissue accumulation, good tumor margin imaging effect, and excellent photothermal conversion effect. Therefore, the IFDG could realize accurate long-term monitoring and photothermal therapy non-invasively of the hepatic tumor to reduce its size. Next, the complete resection of the hepatic tumor in situ lesions could be realized by the intraoperative real-time NIR-II imaging guidance. Notably, the preoperative downstaging strategy is confirmed to lower the postoperative recurrence rate of the liver cancer patients under middle and advanced stage effectively with fewer side effects. Overall, the designed nanoplatform demonstrates great potential as a diagnostic-therapeutic integration platform for precise imaging-guided surgical navigation of orthotopic hepatic tumors with a low recurrence rate after surgery, providing a paradigm for diagnosing and treating the advanced tumors in the future clinical translation application.

**Graphical Abstract:**

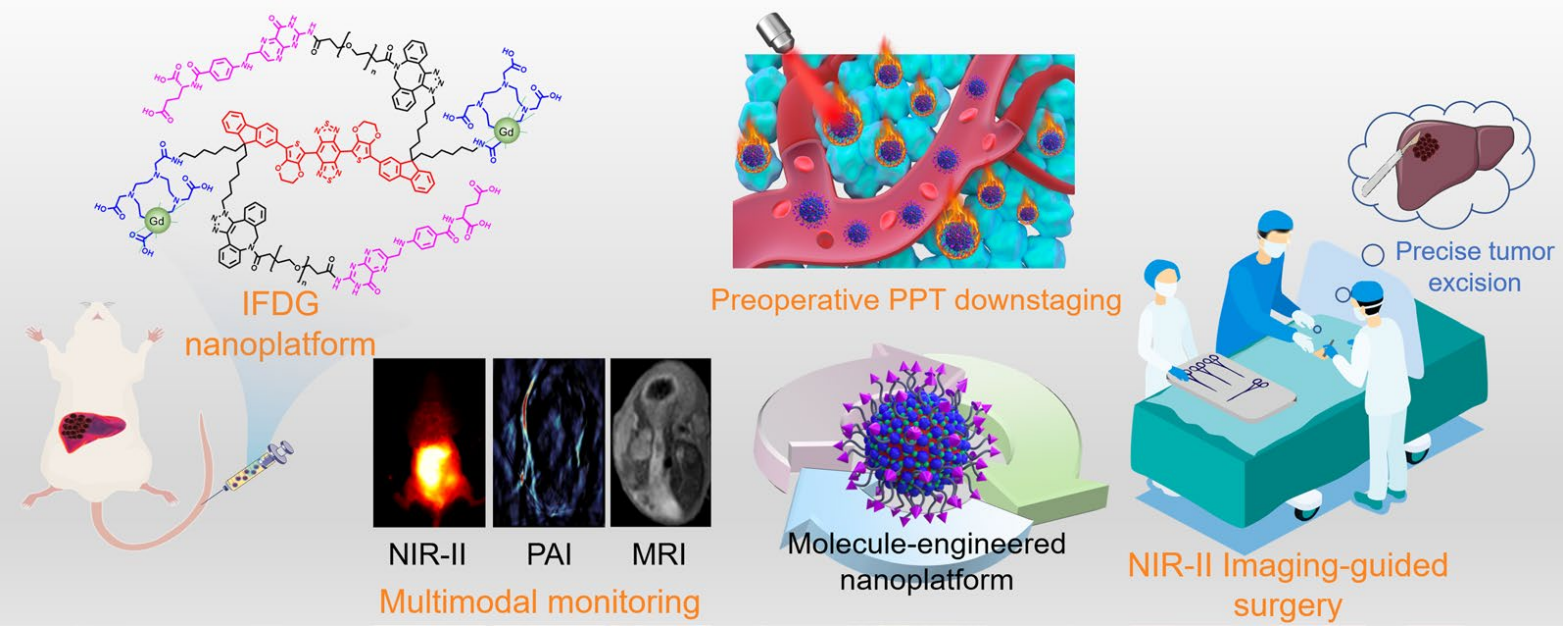

**Supplementary Information:**

The online version contains supplementary material available at 10.1186/s12951-023-02263-w.

## Background

Hepatic tumors are among the most prevalent malignancies encountered in clinical practice, ranking 6th and 3rd in terms of incidence and mortality among malignant tumors [1–3]. Radical surgery remains the primary therapeutic option for achieving long-term survival in hepatic tumor patients [4–6]. However, these patients often face a grim prognosis, with recurrence rates reaching as high as 70% within 5 years after surgery [7, 8]. The primary contributor to these elevated postoperative recurrence rates is the absence of characteristic symptoms during the early stages of hepatic tumors. Accordingly, most liver tumors are diagnosed in their middle and advanced stages with large sizes. Moreover, the tumors have invaded the surrounding blood vessels and metastasized outside the liver, resulting in incomplete surgical resection and intraoperative metastasis [9, 10]. The National Comprehensive Cancer Network (NCCN) guidelines propose that surgical resection should be done after radiotherapy, chemotherapy, immunotherapy, or other approaches to downstage and inactivate the middle and advanced primary hepatic tumors [11]. These techniques have improved surgical resection moderately, while the high recurrence rate during the continuous follow-up processes still haunts doctors and patients. Because the advanced tumor proliferation and invasion activities are not effectively inhibited, the tumor location and boundary cannot be accurately identified during surgery [12]. Hence, developing a new method for precise diagnosis and treatment of the hepatic tumor is urgently needed to improve the resection surgery’s efficiency.

With the development of molecular imaging technology, optical imaging surgical navigation has been widely utilized in detecting residual lesions and metastases in primary hepatic tumors, effectively decreasing the post-surgery recurrence rate [13]. Indocyanine green (ICG), a near-infrared I (NIR-I) fluorescent dye with a spectral range of 700–900 nm, has received approval from the Food and Drug Administration (FDA) for clinical applications and has found successful use in liver surgery [14, 15]. However, ICG imaging is plagued by several limitations, including low spatial resolution, limited penetration depth (< 1 cm), and interference from tissue autofluorescence, all of which hinder the effectiveness of surgical navigation. To address these challenges, Tian et al. developed a multispectral imaging instrument that combines visible light and NIR-I/II region imaging techniques, capitalizing on the tailing effect of ICG. Nevertheless, ICG’s imaging capabilities may not be precise enough to distinguish between benign and malignant tumors, and its relatively high accumulation in normal tissues could result in false-positive signals [16].

NIR-II fluorescence imaging has become a promising research hotspot in tumor diagnosis and treatment [17–20]. NIR-II fluorescence imaging has emerged as a powerful tool for precisely identifying deep-seated lesions, all without the need to expose tumor-bearing regions. This capability is attributed to its exceptional tissue penetration and impressive spatiotemporal resolution [21–23]. Real-time intraoperative navigation therapy, reliant on NIR-II fluorescence imaging to accurately delineate tumor boundaries and guide resection procedures, has garnered significant interest and research focus [24–26]. Fang et al. designed a multimodal probe with NIR-II/PAI/MRI performance for early diagnosis of small-sized hepatic tumors. The designed probe performed non-invasive photothermal ablation for in situ hepatic tumors under the guidance of multimodal imaging, achieving the purpose of downstaging hepatic tumors. However, due to the lack of related research on tumor resection guided by fluorescein surgery, the purpose of comprehensive diagnosis and treatment of hepatic tumors had not been realized in a certain sense [27]. In addition, Liu et al. developed a tumor microenvironment-responsive NIR-II probe DCNPs@Si-omSi-RGD, realizing high-resolution imaging of tumor margins and completing surgical resection of the hepatic tumor under the guidance of NIR-II fluorescence imaging. However, the absence of preoperative tumor suppression may alleviate the high recurrence rate of tumors in clinical practice [28]. Hence, the integration of preoperative downstaging with intraoperative real-time-guided resection of hepatic tumors will not only render previously undetectable lesions definitively resectable but also aid in curbing the high recurrence rates post-surgery, offering a novel clinical treatment approach for middle and advanced orthotopic hepatic tumors.

**Scheme 1:**
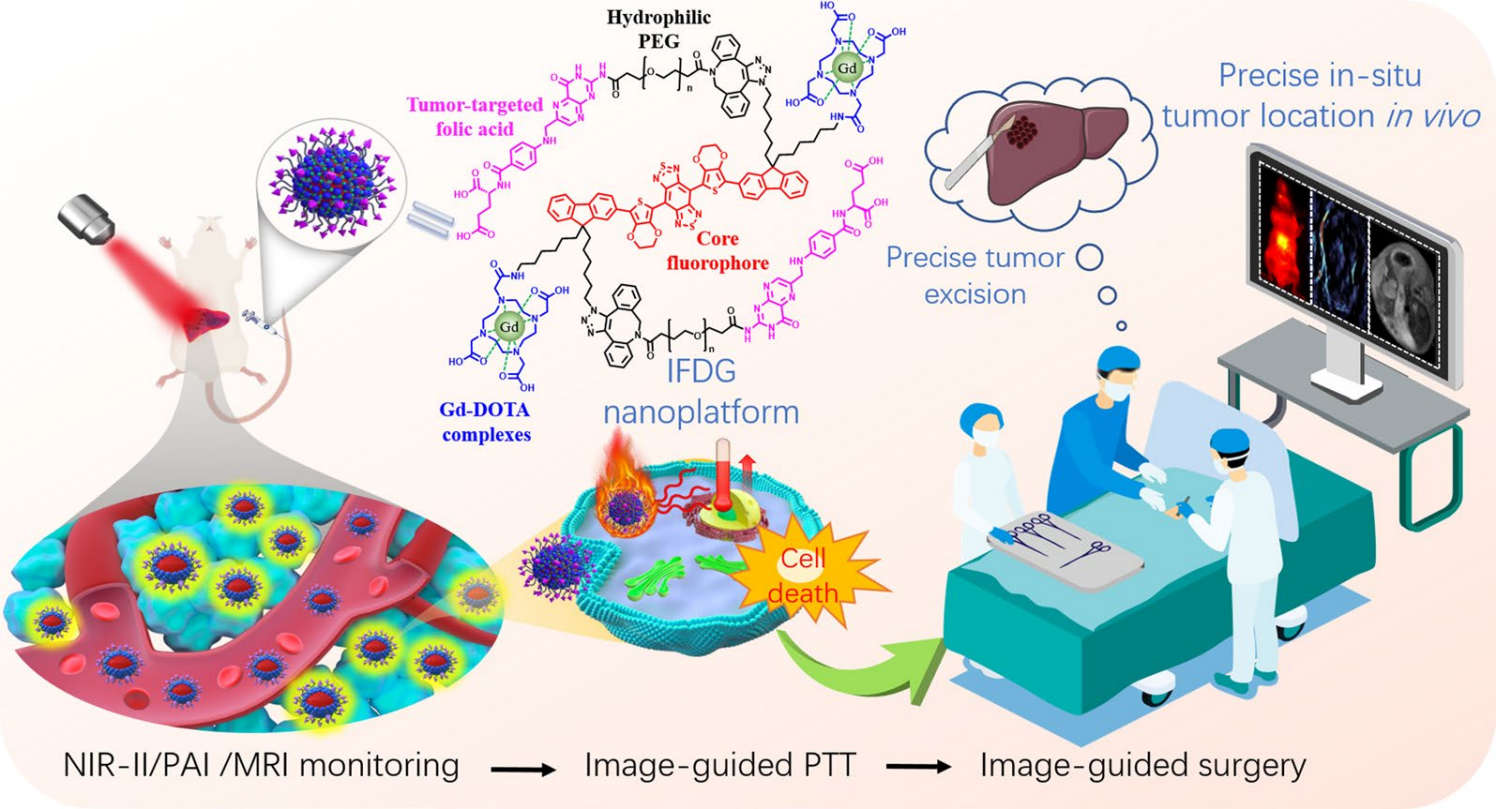
Schematic diagram of imaging monitor, photothermal downstaging therapy, and real-time intraoperative navigation resection of the orthotopic hepatic tumor based on the IFDG nanoplatform

NIR-II organic functional probes have proved to be a superior real-time visualization tool with deeper penetration (∼cm), qualified for fluorescence imaging with good contrast and photothermal therapy with good biocompatibility for tumors. Our group has long been committed to developing multifunctional molecular probes, especially in NIR-II probes [29–33]. In this work, inspired by the “S-D-A-D-S” type NIR-II probe IRFEP developed previously by our group [24, 29], we constructed a NIR-II/PAI/MRI nanoplatform IRFEP-FA-DOTA-Gd (IFDG) for precise diagnosis and therapy of orthotopic hepatic tumors (Scheme 1). The IFDG is fabricated based on the IRFEP with modification of tumor-targeted folic acid (FA) and chelating agent 1,4,7,10-tetraazacyclododecane-N,N′,N″,N‴-tetraacetic acid (DOTA), and then self-assembled into nanoparticle for chelating with Gd agent. The IFDG is confirmed to be competent for early and accurate diagnosis of orthotopic hepatic tumors and subsequent inactivation and downstaging of the tumor by photothermal therapy (PTT). After that, NIR-II intraoperative real-time guided tumor resection is performed to separate the orthotopic hepatic tumor completely. The deep integration of tumor downstaging and resection based on the multifunctional IFDG nanoplatform could improve the negative resection rate of the hepatic tumor in the mid-late stage and realize accurate dynamic monitoring and evaluation after surgery. The above synergistic strategies are confirmed to reduce the rate of postoperative metastasis and recurrence of hepatic tumors, effectively increasing the 5-year survival rate of patients with moderate or advanced hepatic tumor post-surgery in clinical practices.

## Results and discussion

### The characterization of IFDG nanoplatform

**Fig. 1:**
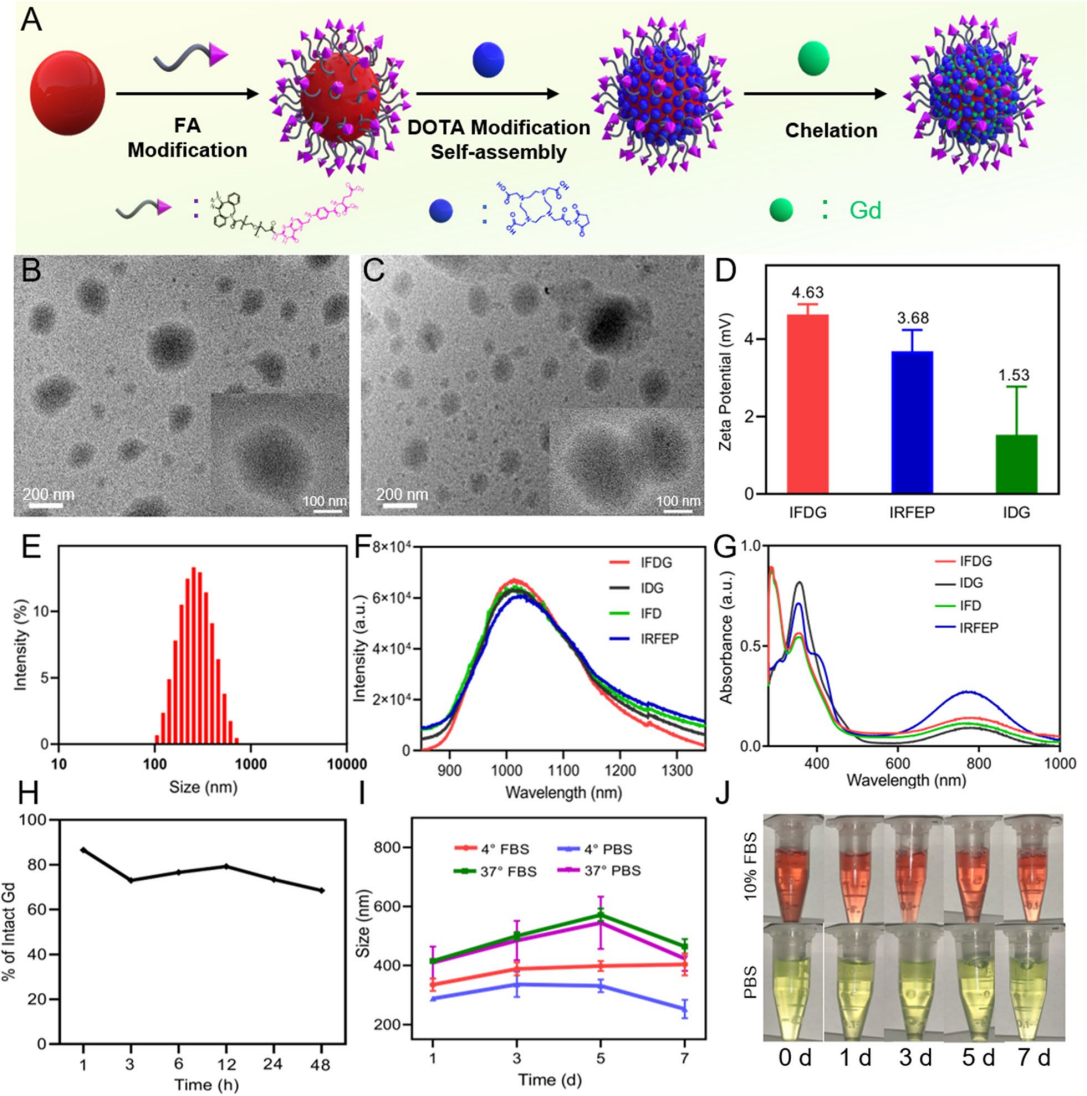
**A** The preparation process of IFDG. **B**, **C** TEM images of IFDG and IDG. **D** Zeta potential of IFDG, IRFEP, and IDG. **E** Hydrodynamic size distributions of IFDG. **F** Fluorescence and **G** UV–vis absorption spectra of IFDG, IRFEP, IFD, and IDG. **H** Gd^3+^ chelation stability of IFDG. **I** Size changes of IFDG under different physiological environments for 7 days. **J** Dispersion of IFDG in different solvents

The IRFEP-FA-DOTA-Gd (IFDG) nanoplatform are prepared by modifying FA, DOTA, and Gd^3+^ agents on the IRFEP molecule (Fig. 1A). The intermediates, including compound 2, compound 5, compound 7, compound 8, compound 9, and IRFEP-FA-DOTA (compound 11), were all characterized by the ^1^H-NMR and ^13^C-NMR spectra (Additional file 1: Figs. S2–S12). The morphology of IFDG and IDG are observed by transmission electron microscope (Fig. 1B, C). The two samples present a sphere-like structure with a similar size distribution. The zeta potentials of IRFEP, IDG, and IFDG are tested to be approximately 3.68, 1.53, and 4.63 mV, respectively (Fig. 1D). The average hydrodynamic size distribution of IFDG was measured to be 255 nm by dynamic light scattering (DLS) (Fig. 1E), which is consistent with the results of TEM. Figure 1F shows the fluorescence spectra of IRFEP, IDG, IFD, and IFDG in the NIR region (850–1500 nm). The results demonstrate that the emission of IFDG has no significant change even after complicated modification and meets the requirements of NIR-II fluorescence imaging. The UV–vis absorption spectra indicate the absorption peaks of the IFDG have a redshift of 5 nm compared with that of the IRFEP (Fig. 1G). The chelation stability of Gd^3+^ in IFDG is confirmed by Gd^3+^ standard relaxation curves (Additional file 1: Fig. S13). The results reveal that the amount of Gd^3+^ chelated in IFDG is still over 65% after 48 h than the initial condition (Fig. 1H), indicating the good chelation stability of Gd^3+^. The stability of IFDG was also investigated by determining the size distribution in PBS, complete medium, and fetal bovine serum at different temperatures for 7 days, respectively. The size distribution and dispersibility change minutely in different physiological environments and temperatures (Fig. 1I, J), indicating that IFDG has favorable stability.

### Imaging and photothermal conversion performance of IFDG

**Fig. 2:**
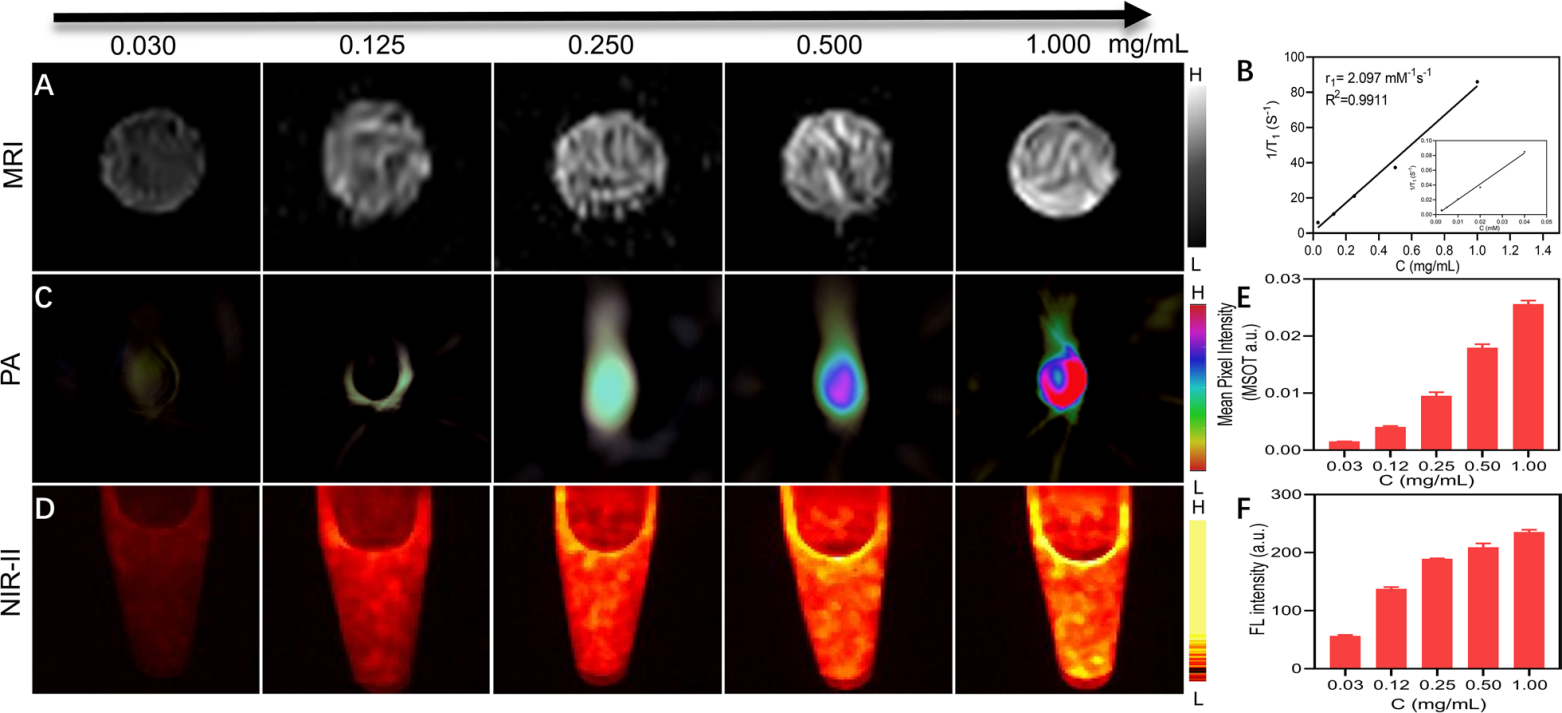
The multimodal imaging performance of IFDG. **A** MRI images of IFDG at different concentrations. **B** T1 relaxation rate curve of IFDG. **C** PA and **D** NIR-II images of IFDG at different concentrations. **E** PA and **F** NIR-II quantification statistics of IFDG

The IFDG has excellent potential to realize reliable multimodal NIR-II/PAI/MRI imaging based on the designed structure. As shown in Fig. 2A, IFDG exhibits effective MR imaging ability according to the relaxivity with a r1 value of 2.097 mM^−1^ s^−1^ (Fig. 2B). As concentration increases, the imaging brightness enhances, and the imaging effect achieves its best at 1 mg/mL. Photoacoustic and NIR-II fluorescence imaging of IFDG presents similar trends with the increased concentration (Fig. 2C, D). The quantification results show that the photoacoustic signal intensity is 0.025 at 1 mg/mL, 17 times that of 0.03 mg/mL, and the fluorescence intensity of 238 is over 4 times higher than that of 0.03 mg/mL (Fig. 2E, F). The above results indicate a significant dose-effect correlation between IFDG concentration and signal intensity of the three imaging models.

**Fig. 3:**
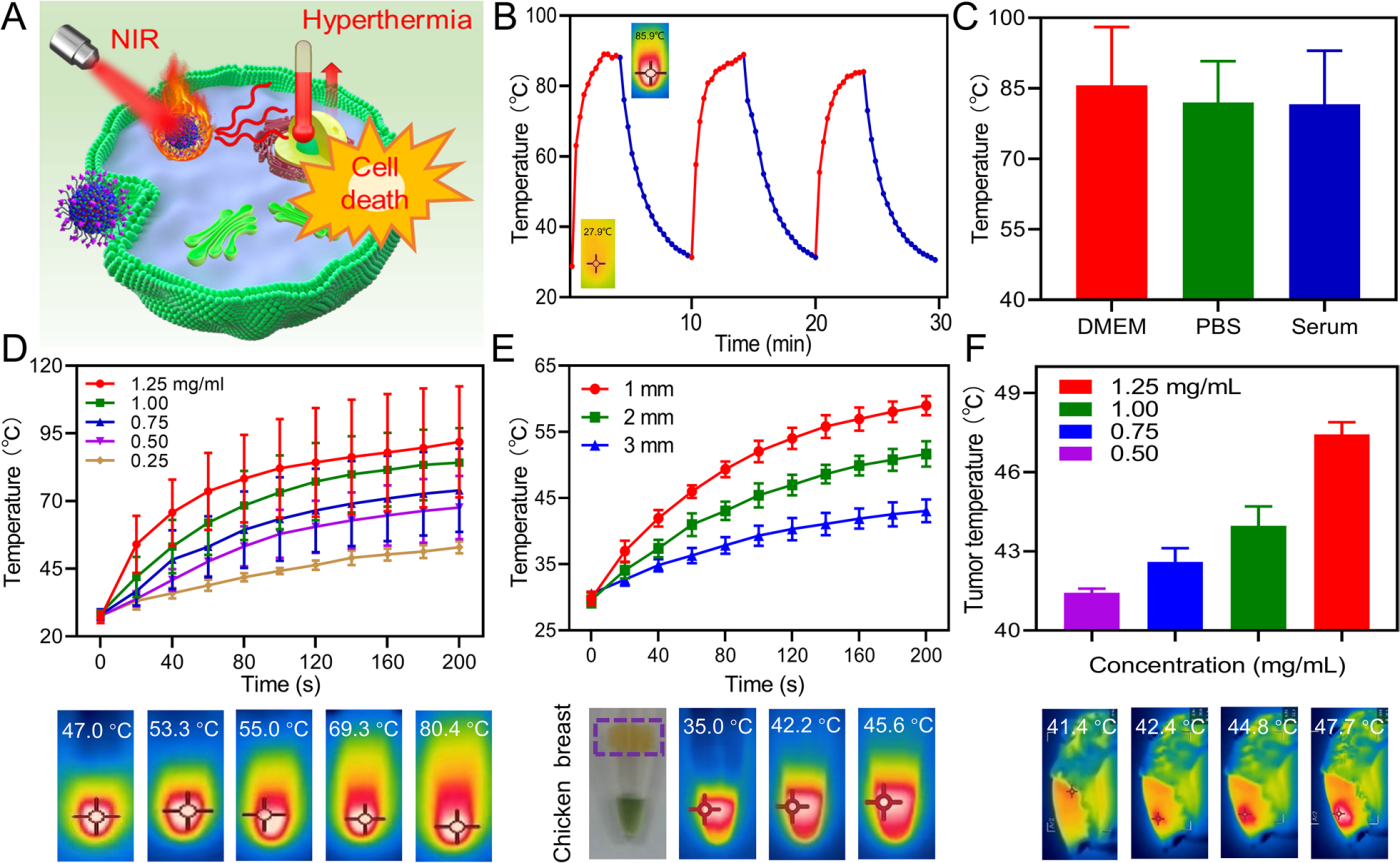
The photothermal effect of IFDG (808 nm, 1.0 W/cm^2^, 10 min). **A** Schematic diagram of the photothermal therapy process of IFDG. **B** Photothermal conversion and stability of IFDG. **C** Temperature of IFDG solution in different solvents after irradiation for 10 min. **D** Photothermal conversion performance of IFDG at different concentrations. **E** The penetration ability of IFDG verified by different thicknesses of biological tissue. **F** Photothermal conversion performance of IFDG at different concentrations under mouse skin

PTT is another essential functionality of IFDG, which play a vital role in downstaging the in situ hepatic tumor (Fig. 3A). Under laser irradiation for 10 min (808 nm, 1 W/cm^2^), the IFDG presented excellent photothermal conversion performance, reaching 86.0 °C under the concentration of 1.25 mg/mL, and good photothermal stability in three “on/off” laser cycles (Fig. 3B). In three different physiological environments (DMEM medium, PBS, and serum), the temperatures of IFDG also achieved 85.6 °C, 82.1 °C, and 81.6 °C, respectively, indicating promising prospects for biological applications (Fig. 3C). The IFDG exhibited concentration-dependent photothermal performance under 808 nm laser irradiation (Fig. 3D, Additional file 1: Figs. S14, S15). Based on the thermal equilibrium temperature and cooling curve, the photothermal conversion efficiency of IFDG is calculated to be 41.5%. Notably, the subcutaneous photothermal effect was also investigated by the biological tissue model. The penetration ability of IFDG was explored in different thicknesses of chicken breast and pork belly. The results indicated that the IFDG possesses a satisfactory laser absorption capacity and generates heat under biological tissue (Fig. 3E, Additional file 1: Figs. S16–S18). As revealed in Fig. 3F, IFDG also exhibited superior photothermal conversion performance under the mouse skin. Under an 808 nm laser irradiation, the subcutaneous temperature could reach 48 °C within 3 min (Additional file 1: Figs. S19, S20). These results suggested that IFDG is propitious for photothermal therapy in vivo.

### In vitro cytotoxicity of IFDG

**Fig. 4:**
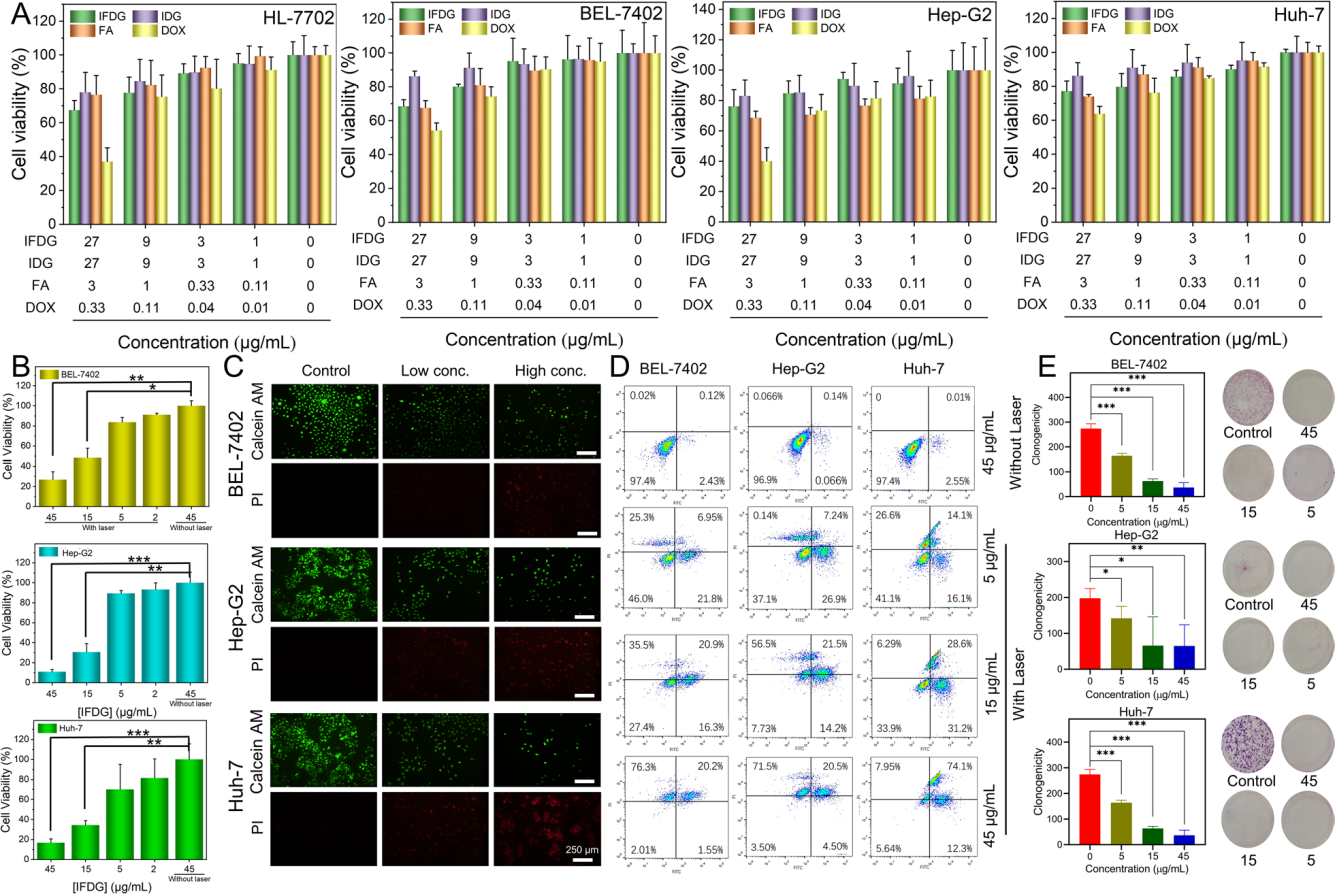
The toxicity and photothermal damage of IFDG to cells. **A** Cytotoxicity of IFDG at different concentrations toward different cell lines. **B** Photothermal cell damage of IFDG (laser: 808 nm, 1.0 W/cm^2^). **C** Live and dead staining results after treatment with different concentrations of IFDG with laser irradiation on liver cancer cell lines. **D** Flow cytometry apoptosis analysis of liver cancer cells after treatment with different concentrations of IFDG with or without laser irradiation. **E** Colony formation results of liver cancer cell lines after treatment of different concentrations of IFDG (*statistically different from the no-laser group, p < 0.05; **significantly different from the no-laser group, p < 0.01; ***extremely different from the no-laser group, p < 0.001)

The cytotoxicity of IFDG, IDG, and FA with different cell lines was examined by the CCK-8 method (8 cultured cell lines, including 5 normal cell lines). IFDG and IDG showed low cytotoxicity and maintained a high cell survival rate at a high concentration (27 µg/mL) (Fig. 4A and Additional file 1: Fig. S21). As a positive control, the doxorubicin (DOX) group with a relatively low concentration presents a noticeable decrease in the cell survival rate.

The photothermal cell damage of IFDG is explored using the BEL-7402, HepG-2, and Huh-7 cell lines (Fig. 4B). With an increase of IFDG concentration, the cell survival rate gradually decreased under laser irradiation. The IFDG groups without laser irradiation always maintained a high survival rate of nearly 100%. The live/dead cell staining results indicated that most cells died in a high concentration of IFDG with the cell morphology changed. The above results showed that IFDG could effectively damage tumor cells under laser irradiation (Fig. 4C). The apoptosis-inducing abilities of IFDG with different treatments were analyzed using the Annexin V-FITC/PI apoptosis kit. As shown in flow cytometry analysis, with the increase of IFDG concentration, the apoptosis rate of the three kinds of cells all increased (Fig. 4D). The apoptosis rate of the IFDG group (45 µg/mL) with laser irradiation (~ 44.4% on average) was significantly higher than that of the group without laser irradiation (~ 1.8% on average). The results of the photothermal colony formation of IFDG are displayed in Fig. 4E. The IFDG exhibited an inhibition effect on the formation of colonies. The fewer cell clusters with increased concentration of IFDG. In contrast, the clonal cell clusters of control groups were distributed evenly and densely. These results proved an excellent photothermal intervention ability of IFDG to the hepatic tumor cells.

### In vitro cell internalization and affinity of IFDG

**Fig. 5:**
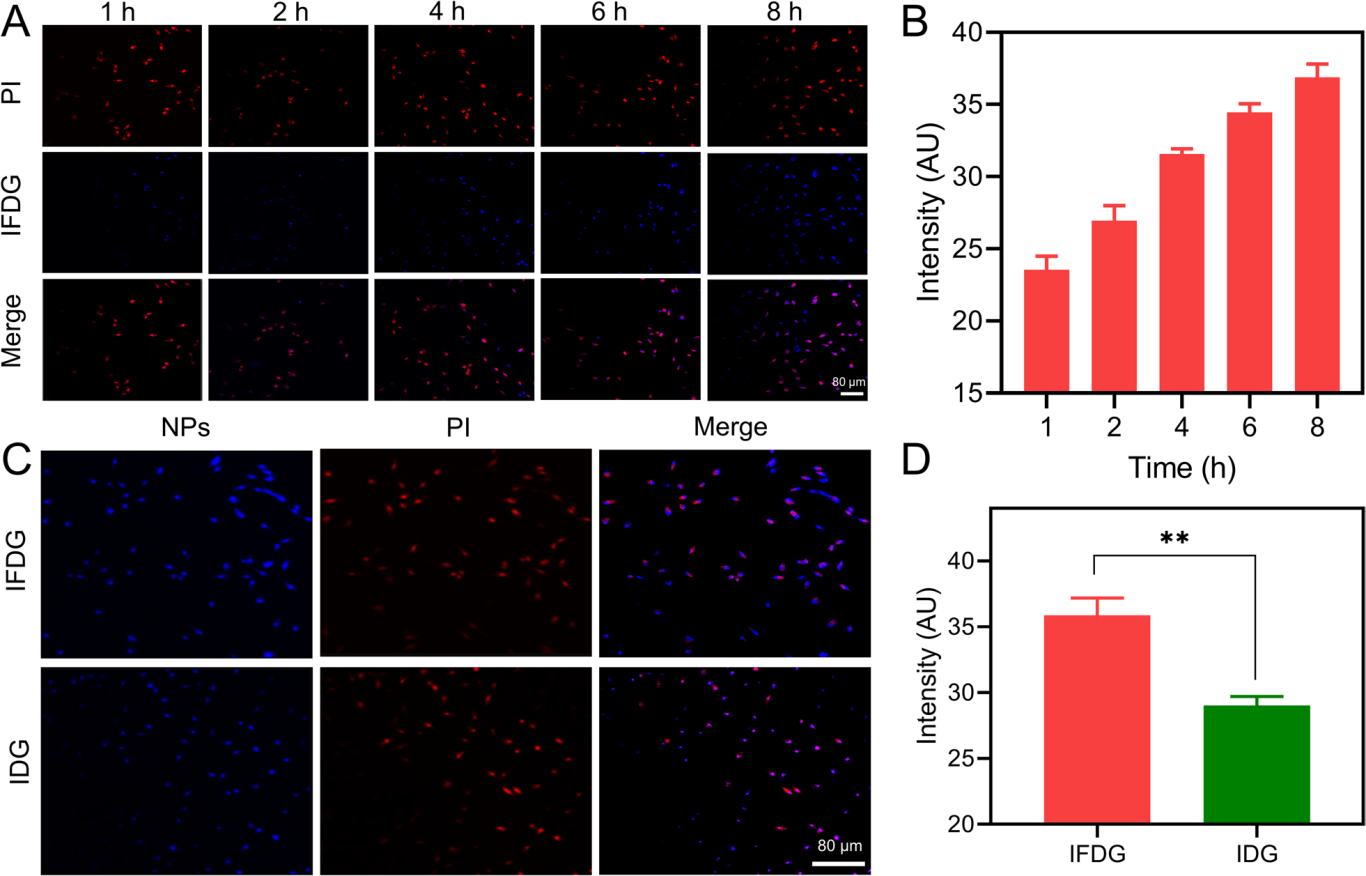
The in vitro cell internalization results of IFDG. **A** Internalization processes of IFDG on the liver cancer cell line BEL-7402 at different time points. **B** Quantitative analysis results of intracellular fluorescence signals of IFDG at different time points. **C** Differences in the accumulation of targeted IFDG and non-targeted IDG on the liver cancer cell line BEL-7402. **D** Quantitative analysis of IFDG and IDG signal intensities in BEL-7402 cells. **Significantly different from the IFDG group, p < 0.01

The cell internalization of IFDG is investigated by laser confocal fluorescence imaging. The imaging and quantitative statistics results presented that IFDG gradually accumulated in the cytoplasm with the increase of time. Remarkably, IFDG fluorescence in the cytoplasm contrasts with the PI-labeled nuclei, meaning that IFDG can effectively internalize into cells and accumulate in the cytoplasm (Fig. 5A, B). In addition, the accumulation amount of IFDG is relatively more than that of IDG, proving that IFDG has a better targeting and internalization effect on hepatic tumor cells within the same period (Fig. 5C, D).

### Biocompatibility in vivo of IFDG

**Fig. 6:**
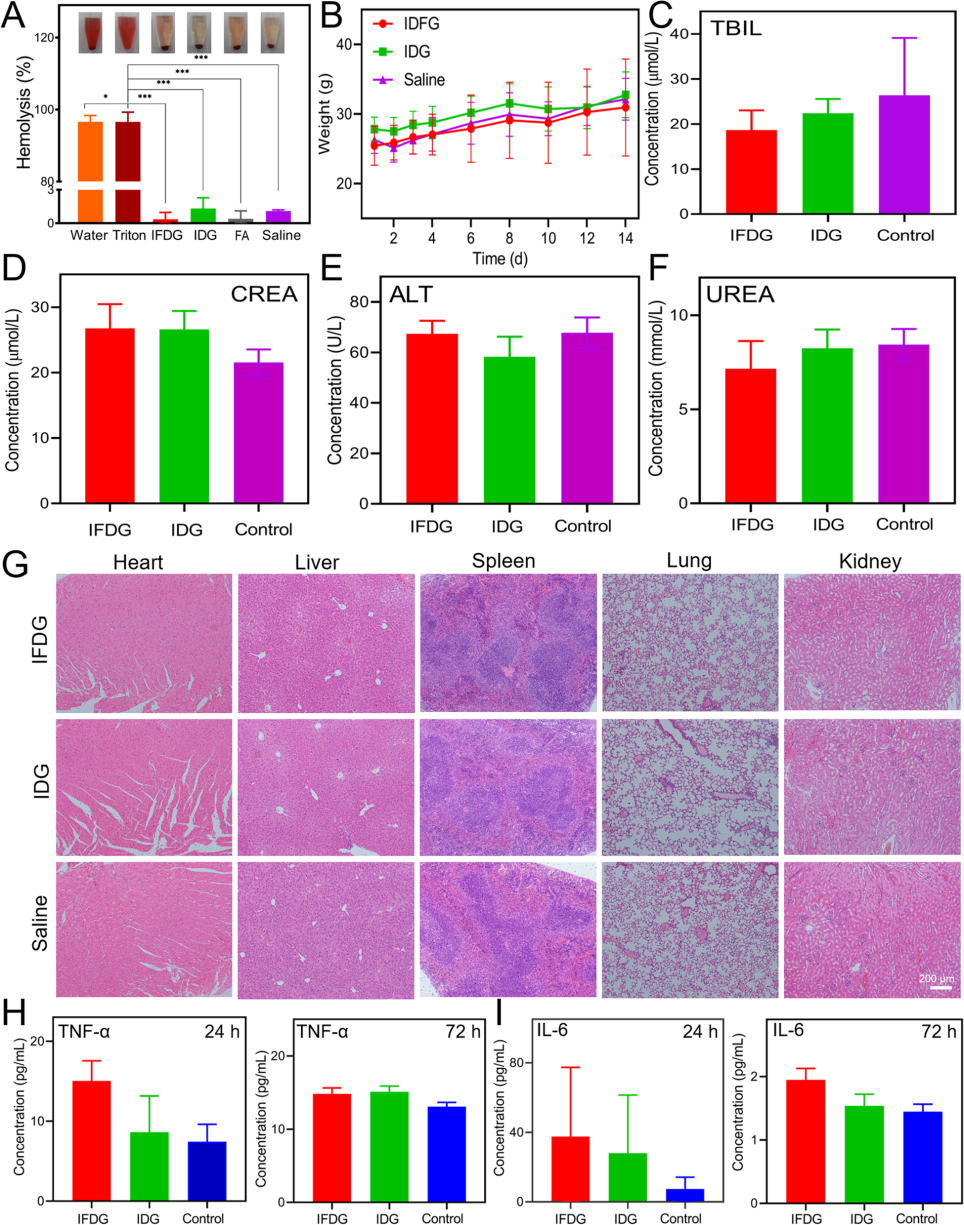
The in vivo biocompatibility of IFDG. **A** Hemolysis results of IFDG. **B** The changes in mice’s body weight during the acute toxicity test. **C**–**F** Blood biochemical analysis of mice after IFDG injection, n = 3. **G** Pathological analysis of major organ tissues with acute toxicity after 14 days. **H**, **I** Major inflammatory factors (IL-6 and TNF-α) test results of mice 24 and 72 h after IFDG injection (*statistically different from the triton group, p < 0.05; ***extremely different from the triton group, p < 0.001)

The hemolysis analysis exhibits that IFDG, IDG, and FA did not cause significant damage to red blood cells and were lower than the safety limit of 3%, indicating the safety of IFDG for injection intravenously (Fig. 6A). The acute toxicity of IFDG and IDG was assessed by the BALB/c mice model. As displayed in Fig. 6B and Additional file 1: Fig. S22, the mice survived well with the treatment of IFDG and IDG, and the adverse symptoms were relatively mild. The body weight of the mice all increased after 14 days, denoting the mice were well-developed after injection. The results of the blood biochemical analysis indicate that most indicators had no significant difference among the mice injected with IFDG, IDG, and saline. Only the TBIL count in the treatment group was significantly lower than the saline group, which may be related to their effects on liver metabolism (Fig. 6C–F and Additional file 1: Fig. S23). The pathological results of the mouse organs showed no obvious pathological changes were found in the major organs between the treatment and control groups, except a small number of lymphocytes infiltrated the liver in the treatment group (Fig. 6G). As shown in Fig. 6H, I, the levels of inflammatory factors IL-6 and TNF-α in treatment groups were relatively higher than that of the control group after 24 h. However, after 72 h of in vivo metabolism, there was no significant difference in the levels of inflammatory factors among all groups. These results demonstrate that IFDG and IDG have satisfactory biocompatibility in vivo.

### In situ multimodal imaging of hepatic tumor in vivo based on IFDG

**Fig. 7:**
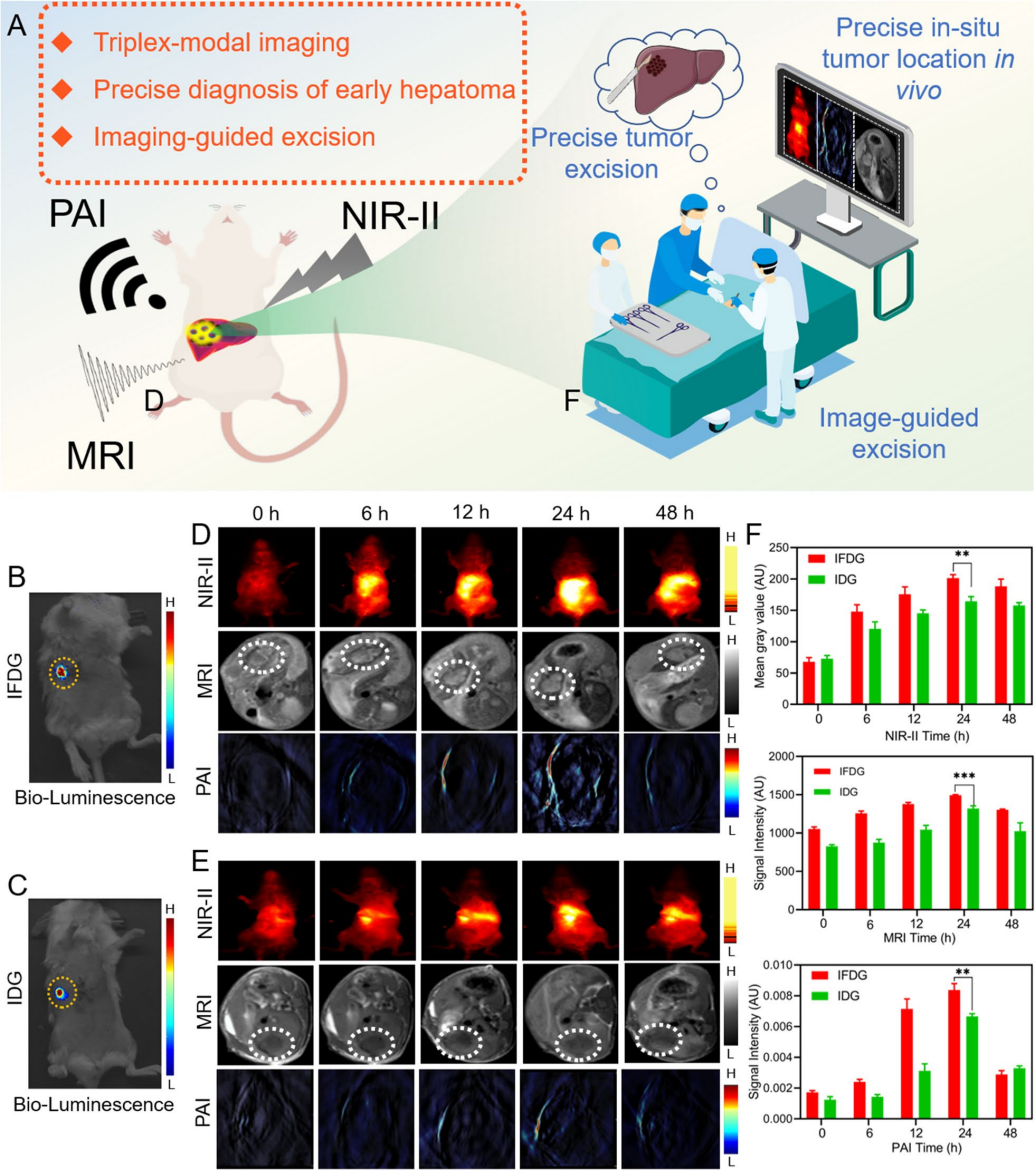
The in vivo imaging of IFDG. **A** Schematic diagram of tumor multimodal imaging surveillance. Tumor location of orthotopic liver cancer tumor modeling of **B** IFDG and **C** IDG group mice. NIR-II, MR, and PA imaging results of mice at different time points after **D** IFDG and **E** IDG injection, n = 3. **F** Quantitative analysis of NIR-II, MR, and PA imaging signal in the liver region at different time points (**significantly different from the IFDG group at 24 h, p < 0.01. ***extremely different from the IFDG group at 24 h, p < 0.001)

The IFDG is designed to distinguish the tumor and normal tissue based on its multimodal imaging functions. The mouse subcutaneous/orthotopic hepatic tumor model is investigated for imaging monitoring and subsequent precise excision (Fig. 7A). Most theranostics studies on the hepatic tumor focused on the ectopic subcutaneous tumor model [24, 34–37], thus lacking sufficient evidence for treating the orthotopic hepatic tumor. Animal orthotopic hepatic tumor models are closer to real clinical application scenarios than the ectopic subcutaneous tumor model, providing great significance in transforming clinical application. To this end, we focused on establishing an orthotopic hepatic tumor mouse model and evaluating the effects of IFDG imaging monitoring, photothermal downstaging therapy, and imaging-guided surgery on this model. The orthotopic tumor locations in mice are shown in Fig. 7B, C, which were captured by the bioluminescence imaging. Then, the IFDG and IDG were intravenously injected into the grouped mice, respectively for evaluating their imaging monitoring performance on the orthotopic hepatic tumor model (Fig. 7D, E). After 24 h of tail vein injection, the NIR-II/PAI/MRI signal of the liver tumor site in orthotopic hepatic tumor mice reached their peaks. The outline and edge of the tumor were displayed in MRI and NIR-II imaging, and the small lesions in the liver can also be found in the IFDG group. Compared with the IDG group, the imaging signals of the IFDG group relatively enhanced owing to the tumor-targeting effect of the FA according to the quantitative statistical results of the three imaging models (Fig. 7F) [38–41]. The imaging performances of IFDG and IDG in the subcutaneous tumor model were also explored. The results were highly consistent with those of the orthotopic tumor model (Additional file 1: Fig. S24–S26). The three imaging methods present clear signals in the tumor area after 6 h and peak after 24 h with a strong signal within 48 h. In addition, organs/tumor images and corresponding quantitative statistics results indicated the excellent accumulation of IFDG in tumor tissues (Additional file 1: Figs. S27, S28). The above results showed that IFDG could be employed for real-time monitoring of hepatic tumor in situ tumors.

### Photothermal downstaging and imaging-guided resection of orthotopic hepatic tumor

**Fig. 8:**
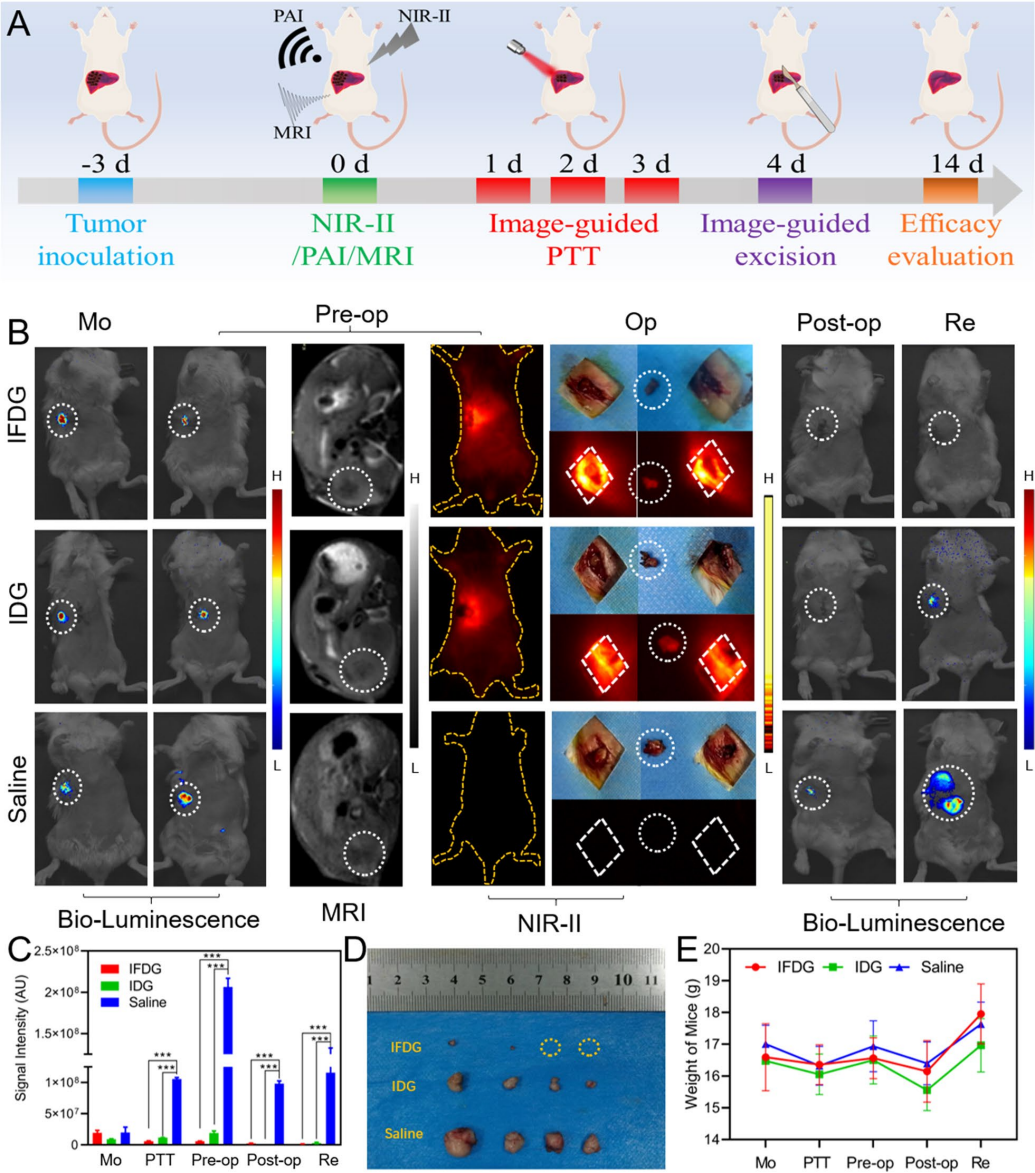
The in vivo tumor photothermal downstaging and imaging guided surgical resection of IFDG. **A** Schematic diagram of the construction of the orthotopic hepatic tumor models. **B** The mice imaging results after treatment with IFDG, IDG, and normal saline in the stage before and after downstaging, pre-operation, operation, post-operation, and recurrence monitoring, respectively. **C** Quantitative analysis of tumor site IVIS signals in different groups of mice (**significantly different from the saline group, p < 0.01; ***extremely different from the saline group, p < 0.001). **D** Digital photos of tumors. **E** Body weight change curves of the mice

The tumor area could be precisely recognized based on the accurate imaging results [19]. Thus, we accomplished photothermal downstaging and imaging-guided excision therapy in the orthotopic hepatic tumor model in vivo (Fig. 8A). A total of 12 mice with orthotopic hepatic tumors were selected for the in vivo anti-tumor therapy. Before photothermal downstaging treatment, the imaging signals of the tumors in all groups were at the same level. As the tumor proliferated and deeply invaded the surrounding liver tissue, surgical resection became more and more difficult. Conspicuously, the imaging performance of the IFDG group was better than that of the IDG group. The IFDG group has strong signals, high brightness, a clear range, and clear indications, while the IDG group obtains blurred boundaries (Fig. 8B). The downstaging intervention was a critical point of this research. After 4 days of the intervention of photothermal therapy, the tumor signals of both the IFDG group and the IDG group dropped, and the therapeutic effect of the IFDG group was better than that of the IDG group (Additional file 1: Figs. S29, S30). After the precise positioning evaluation with the preoperative MR and injection with the designed nanoplatforms, the intraoperative NIR-II fluorescence imaging were applied in the surgical resection at the peak signal of the liver tumor area after 24 h. The surgical resection process with different treatments was presented in Additional file 1: Fig. S31.

In the postoperative and recurrence monitor stage, the IFDG group had tiny signals in the original tumor site averagely after treatment. In contrast, all mice in the IDG and control group had recurred with relatively obvious signals (Fig. 8C). After the mice were euthanized, all the tumor and organ tissues were taken out. As displayed in Fig. 8D, only two tiny tumors were found in the IFDG group, while both the IDG and control groups had relatively big tumors. From the body weight changes data, it is found that the body weight of the mice in the three groups decreased significantly and then continued to increase 5 days after the operation. In addition, the average mice body weight change of the IFDG group was more than that of IDG and control groups during this period. The body weight increase in the control group may be due to the rapid growth of the tumor (Fig. 8E). These results indicated that the IFDG has an excellent photothermal intervention for the orthotopic hepatic tumor, which could be completely resected under the guidance of NIR-II with an improved negative resection margin of the tumor, reducing the recurrence rate of the tumor after surgery.

## Conclusions

In summary, we developed a novel NIR-II/PAI/MRI multifunctional organic molecular nanoplatform IFDG for imaging monitor, photothermal downstaging, and imaging-guided surgical resection of orthotopic hepatic tumor. The modification of FA enhances the accumulation amount of IFDG in the tumor cell, improving the diagnosis and treatment efficiency of hepatic tumor. After preoperative photothermal therapy, the hepatic tumors with relatively large volume are down-staged to reduce the size. Then, the intraoperative NIR-II imaging-guided resection precisely remove the small and imperceptible residual tumor tissue with a low recurrence rate. This work provides a comprehensive and effective collaborative strategy for solving the clinical problem of the high 5-year recurrence rate of hepatic tumor. Our subsequent work will further explore whether the new multimodal nanoplatform has long-term chronic metabolic toxicity, organ damage, and reproductive toxicity, building a solid foundation for future clinical transformation.

## Materials and methods

### Materials

DBCO-PEG_2000_-FA was purchased from Pengsheng Biotechnology Co., Ltd. DOTA-NHS was purchased from Xi’an Ruixi Biotechnology Co., Ltd. Silica gel and silica gel plates were purchased from Qingdao Ocean Chemical Engineering Co., Ltd. Lidocaine, propidium iodide, and sodium hydroxide were purchased from Shanghai Sigma Co., Ltd. Gadolinium (III) chloride hexahydrate (GdCl_3_ 6H_2_O) was purchased from Beijing Bailingwei Technology Co., Ltd. CCK-8 kit was purchased from Shanghai Macklin Co., Ltd. Folic acid (FA) and doxorubicin (DOX) were purchased from Shanghai Aladdin Co., Ltd. Calcein/PI Cell Viability Detection Kit, Annexin V-FITC/PI Cell Apoptosis Detection Kit, IL-6 ELISA Detection Kit, TNF-α ELISA Detection Kit, and Colony Formation Staining Solution were purchased from Shanghai Beyotime Biotechnology Co., Ltd. Isoflurane was purchased from Shenzhen Ruiwode Life Technology Co., Ltd. Medical surgical glue was purchased from Beijing Shunkang Technology Development Co., Ltd. Cell culture medium, trypsin, ampicillin, and streptomycin sulfate were purchased from Hyclone Company. Human liver cancer cell lines (BEL-7402, Hep-G2, and HuH-7) and normal cell lines (HL-7702, HT-22, L929, IMR90, and HEK293) were all derived from American type culture collection (ATCC).

### Synthesis and characterization of IRFEP-FA-DOTA

Firstly, 2-bromofluorene (6.0 g), 1,6-dibromohexane (59.3 g), and tetrabutylammonium bromide (0.78 g) were added to 150 mL potassium hydroxide aqueous solution (45%) and then stirred for 1 h. **Compound 2** could be obtained after purification. Then, 2.13 g of **compound 3** was dissolved in 25 mL of tetrahydrofuran solution, and 7.2 mL of *n*-butyllithium solution was added. After 2 h, 5.86 g of tributyltin chloride was added and stirred overnight to obtain **compound 4**. Afterward, 5.71 g of compound 2 and compound 4 were dissolved in 100 mL of xylene, and 1.16 g of tetrakis triphenylphosphine palladium was added and stirred for 3 h to obtain **compound 5**. After that, 1.9 g of compound 5 was dissolved in 15 mL of tetrahydrofuran solution, 1.80 mL of n-butyllithium solution, and 1.46 g of tributyltin chloride were added with stirring overnight to obtain **compound 6**. Then, 352 mg of compound BBTD and compound 6 were dissolved in 20 mL of xylene, and 70 mg of bistriphenylphosphine palladium dichloride was added and stirred to obtain IR-FE (**compound 7**).

Then, 50 mg IR-FE and 47 mg sodium azide were dissolved in 3 mL DMF and heated for 5 h to obtain IRFE-N_3_ (**compound 8**). Subsequently, 20 mg of compound 8 was dissolved in 2 mL of DMF, 38 mg of DBCO-PEG_2000_-FA, and 20 mL of methyl tert-butyl ether were added to obtain the target product IRFEP-FA-N_3_ (**compound 9**). 20 mg of compound 9 and triphenylphosphine were added to a Schlenk bottle, injected into 2 mL of degassed DMF, stirred at room temperature for 10 h, and placed in an ice-water bath to obtain IRFEP-FA-NH_2_ (**compound 10**). Compound 10 from the previous step was added to 2 mL of DMF, and 10 mL DOTA-NHS (dissolved in tetrahydrofuran, 2 mg/mL) was dripped into the above solution. Then, after stirring for 24 h, the obtained products were lyophilized to yield 12 mg IRFEP-FA-DOTA (**IFD**, **compound 11**) (Additional file 1: Fig. S1). Compound 11: ^1^H NMR (500 MHz, DMSO-d6) δ 11.57 (s, 2H), 8.71 (s, 2H), 8.48–8.26 (m, 1H), 8.15–7.81 (m, 8H), 7.81–7.61 (m, 6H), 7.65–6.83 (m, 15H), 6.78–6.44 (m, 5H), 4.73–4.11 (m, 10H), 3.57 (s, 374H), 2.98–2.74 (m, 6H), 2.38–1.85 (m, 14H), 1.23–1.04 (m, 29H), 0.77–0.43 (m, 2H). ^13^C NMR (125 MHz, DMSO) δ 128.42, 111.63, 72.54, 70.25, 70.04, 69.55, 50.92, 49.20, 46.37, 28.48, 27.30, 25.72.

In addition, IRFEP-DOTA was also synthesized as the following method. Compound 8 (50 mg) was dissolved in 10 mL THF. 10 mg CuTc, 70 mg PEG2000, and 3 mg TBTA was added and stirred for 3 h to obtain IRFEP-N_3_ (IRFEP). 50 mg IRFEP-N_3_ and triphenylphosphine were added to a Schlenk bottle. The solution was stirred for 10 h and then placed in ice water to obtain IRFEP-NH_2_. DOTA-NHS (70 mg) solution was dropwise added. The solution was stirred for 24 h and lyophilized to obtain IRFEP-DOTA.

### Preparation of IRFEP-FA-DOTA-Gd nanoplatform

First, 1 mg IRFEP-FA-DOTA was dissolved in 1 mL of deionized water. The solution was dialyzed for 3 h under stirring with a 1 kDa dialysis bag. Then, before adding 200 µL of GdCl_3_ solution (10 mg /mL), the pH of the solution was adjusted to 7 with NaOH solution (0.1 mol/L). After shaking for 3 h, the solution was treated with a PD-10 column to obtain IRFEP-FA-DOTA-Gd (IFDG). Similarly, IRFEP-DOTA-Gd (IDG) was prepared by the above processes except for replacing IRFEP-FA-DOTA (IFD) with IRFEP-DOTA (IPD).

### Characterization of IRFEP-FA-DOTA-Gd nanoplatform

The particle size and Zeta potential distribution of the nanomedicine IFDG were measured using a Malvern particle size analyzer (Nano-ZS90, Malvern, UK). The morphology of IFDG was observed using a TEM (JEM-2100Plus, JEOL, Japan).

GdCl_3_ was dissolved in ultrapure water and diluted at different times to draw the relaxation curve. The relaxation rates of IFDG recorded at different time points were used to evaluate the Gd^3+^ chelation stability of IFDG. IFDG was dispersed in saline and cell culture medium, which simulated different physiological environments to evaluate the colloidal stability. The ultraviolet absorption curves of IRFEP, IDG, IFD, and IFDG were measured using a UV-visible spectrophotometer (Hitachi, Japan, TU-1900). The fluorescence spectra of IRFEP, IDG, IFD, and IFDG were measured using a steady-state-transient fluorescence spectrometer (Edinburgh Instruments, UK).

Imaging performance tests of the IFDG nanoplatform were conducted as follows: the IFDG solution was diluted to different concentrations. A 0.5 T nuclear magnetic resonance imager (MesoMR-00 H-I, Shanghai Numei, China), MSOT Invision photoacoustic imaging system (MSOT 128, iTheraMedica, Germany), and NIR-II area small animal imaging system (Series II 900/1700-H, Suzhou Yingrui, China) were used to obtain the magnetic resonance imaging, photoacoustic imaging, and near-infrared imaging results of IFDG.

### Photothermal conversion performance of IFDG nanoplatform

IFDG and IDG solutions (1.25 mg/mL) were irradiated with a laser (808 nm, 1.0 W/cm^2^) until reaching the equilibrium temperature. Then, the laser was turned off, and the solution returned to ambient temperature gradually. The on/off cycle was performed three times to verify the photothermal stability of IFDG. The temperature changes were recorded with a thermal imager (FLIR, E4-XT, USA). Lasers with the same parameters were used to irradiate solutions with different IFDG concentrations to evaluate the photothermal conversion effect of IFDG. Chicken and pork bellies with different thicknesses were used to simulate biological tissues to test the light-to-heat conversion effect of IFDG. BALB/c mice were subcutaneously injected with different concentrations of IFDG and IDG solutions. The injection site was irradiated with laser light to evaluate the photothermal conversion effect in vivo.

The photothermal conversion efficiency (*η*) could be calculated by the following equation:

1 $${\eta}=\frac{{hS}({T}_{\max}-{T}_{surr})-{Q}_{\text{dis}}}{I(1-10^{{-A}_{808}})}$$

where *h* represents the heat transfer coefficient, *S* denotes the surface area of the sample container, *T*_max_ denotes the maximum steady-state temperature, *T*_surr_ denotes the ambient room temperature, *Q*_dis_ denotes the heat dissipated by light absorption, *I* denotes the laser power, and *A*_808_ denotes the absorbance of PdMo bimetallene at 808 nm.

### In vitro toxicity and photothermal therapy

Three hepatic tumor cell lines (BEL-7402, Hep-G2, Huh-7) and five normal cell lines (HL-7702, HT-22, L929, IMR90, HEK293) were cultured to logarithmic phase and seeded into 96 wells plate. The cells were treated with IFDG, IDG, and FA in a concentration gradient and DOX as a positive control. After incubating for 72 h, the cells were treated with a CCK-8 test to calculate the cell viability.

Hep-G2, BEL-7402, and Huh-7 cells cultured in 96-well plates were treated with different concentrations of IFDG and then irradiated with a laser (808 nm, 1.0 W/cm^2^) for photothermal cell damage and colony formation assays. Finally, the cells cultured in 24-well plates were incubated with different concentrations of IFDG and irradiated with lasers with the same parameters. The in vitro photothermal killing effect of IFDG on hepatic tumor cell lines was then detected by Calcein/PI cell staining kit.

Hep-G2, BEL-7402, and Huh-7 cells cultured to the logarithmic growth phase were incubated with IFDG, irradiated with a laser (808 nm, 1.0 W/cm^2^), and tested by flow cytometry (S3e, Bio-Rad, US) after apoptosis staining.

### In vitro cell delivery performance tests

To confirm that IFDG can effectively enter and accumulate in hepatic tumor cells, we cultured BEL-7402 cells in confocal culture dishes. When they grew to 50% of density, the cells were added to IFDG solution (1 mg/mL) and incubated for different time lengths. The nuclei were labeled with PI after Paraformaldehyde fixation. A laser confocal microscope (Olympus Co., Ltd., System FV3000) was used to observe the internalization of IFDG. To verify the FA-mediated internalization, IFDG, and IDG groups were set up for comparative observation, respectively.

### In vivo biocompatibility tests

IFDG was dispersed into 2% rat erythrocyte suspension and incubated at 37 °C for 2 h. After centrifugation (8000 rpm, 3 min, 4 °C), the supernatant was collected for testing. Absorption wavelength at 545 nm was measured to calculate hemolysis rates.

Thirty BALB/c mice (half male and half female with an average body weight of 20 g) were randomly divided into three groups: IFDG, IDG, and saline, respectively, and intravenously injected with IFDG (7.5 mg/kg) for acute toxicity test. The mice were observed continuously for 14 days. The signs were recorded, and the survival rate was calculated. Finally, the mice were euthanized, and the major organs were collected for the pathological section. Twenty-seven BALB/c mice were randomly divided into three groups as before arrange and used for analysis of blood biochemistry and inflammatory factors. Three mice in each group were randomly selected to collect venous blood 24 h after injection. The samples were sent for testing of blood biochemistry which included alanine aminotransferase (ALT), aspartate aminotransferase (AST), albumin (ALB), and urea (UREA), creatinine (CREA), creatine kinase (CK), lactate dehydrogenase (LDH), and total bilirubin (TBIL). In analysis of inflammatory factor, three mice in each group were randomly selected to collect serum at 24 and 72 h after injection, respectively, to assess the levels of interleukin-6 (IL-6) and tumor necrosis factor (TNF-α), to define whether IFDG and IDG would cause a systemic inflammatory response.

### Orthotopic and subcutaneous hepatic tumor models establishment

Male BALB/c-nu/nu nude mice were used for subcutaneous tumor formation. BEL-7402 Luc cell suspension was injected into the crotch of each nude mouse at a dose of 100 µL/mouse. The subsequent experiments were conducted after the subcutaneous tumor grew to 6 × 6 mm^3^.

Male SCID mice were used to establish an orthotopic hepatic tumor model. The subcutaneous tumor was dissected in advance. The tumor mass was cut to about 1 mm with a scalpel, soaked in pure medium, and placed in an ice box for later use. The mice were implanted with tumor mass in the liver under anesthesia. The wound was sutured and disinfected. Lidocaine was given for local analgesia. The state of the mice was closely observed, and the wounds were disinfected daily. The modeling was evaluated by IVIS system via bioluminescence in vivo.

All animal experiments were approved by the University of South China Animal Experiment Ethics Review and the Health Guide for the Care and Use of Laboratory Animals of National Institutes. All mice received care by international ethics guidelines.

### In vivo hepatic tumor monitoring

The subcutaneous and orthotopic hepatic tumor mice were divided into IFDG and IDG groups. Nanoplatforms (0.75 mg/mL, 200 µL/mouse) were injected into the mice intravenously. Magnetic resonance images and signal intensities data of tumor sites were collected at 0 h, 6 h, 12 h, 24 h, and 48 h, respectively. T1WI (TR = 500 ms, TE = 11 ms, Fov = 50 × 50 × 22 mm, Voxel = 0.25 × 0.25 × 1.5 mm, matrix = 200 × 192 × 15).

Photoacoustic imaging experiments on the selected model mice were conducted with MSOT invision photoacoustic imaging system. The sampling time intervals were the same as before.

The near-infrared II regions fluorescence imaging experiment was carried out using the near-infrared II regions small animal live imaging system. The sampling time intervals are the same as before. After imaging, the mice were euthanized. The heart, liver, spleen, lung, kidney, and tumor were collected, and the imaging signals of these organs were detected under the same conditions.

### In vivo photothermal therapy and near-infrared surgical guided resection

Twelve orthotopic hepatic tumor model mice were randomly divided into IFDG, IDG, and control groups. The tumor site was located by bioluminescence imaging. The samples were intravenously injected 24 h before the intervention. The tumor site was detected by MRI and NIR-II imaging and irradiated intermittently with a laser (808 nm, 1.0 W/cm^2^). Tumor growth was continuously monitored by the IVIS imaging system, and body weight changes were continuously recorded for the following surgery.

The corresponding samples were injected intravenously into the mice 24 h before the surgery. After the preoperative multimodal imaging confirmed the tumor location, the mice were anesthetized with isoflurane. The tumors were resected under the guidance of near-infrared light imaging. The vital signs of the mice were monitored, and the body temperature of the mice was kept stable throughout the process. After resection, the incision site was sutured and disinfected layer by layer. Lidocaine was treated dropwise for analgesia. The mice were closely observed for 2 h after awakening from anesthesia and placed in an SPF environment for 3 days.

After the surgery, the signs and body weight of the mice were monitored and recorded daily. The mice were given appropriate nutritional support, and the wounds were kept clean and infection free. On the 10th day after the surgery, IVIS and MRI were performed to evaluate the tumor recurrence in the three groups of mice. The mice were then euthanized by carbon dioxide inhalation, and the tumor tissues were collected.

### Supplementary Information

**Additional file 1: Figure S1.** Schematic diagram of the synthetic route of IRFE-PEG-FA-DOTA-Gd. **Figure S2.** The hydrogen spectrum of compound 2. **Figure S3.** The carbon spectrum of compound 2. **Figure S4.** The hydrogen spectrum of compound 5. **Figure S5.** The carbon spectrum of compound 5. **Figure S6.** The hydrogen spectrum of compound 7. **Figure S7.** The carbon spectrum of compound 7. **Figure S8.** The hydrogen spectrum of compound 8. **Figure S9.** The carbon spectrum of compound 8. **Figure S10.** The hydrogen spectrum of compound 9. **Figure S11.** The hydrogen spectrum of compound 11 (IRFEP-FA-DOTA). **Figure S12.** The carbon spectrum of compound 11 (IRFEP-FA-DOTA). **Figure S13.** Gd^3+^ relaxation curves. **Figure S14.** Images and quantitative analysis images of IFDG in different concentrations under laser irradiation (808 nm, 1.0 W/cm^2^, 4 min). **Figure S15.** Images and quantitative analysis images of IDG in different concentrations under laser irradiation (808 nm, 1.0 W/cm^2^, 4 min). **Figure S16.** Verification laser penetration ability images and quantitative analysis images under different thicknesses of pork belly (808 nm, 1.0 W/cm^2^, 4 min, IFDG concentration 1.25 mg/mL). **Figure S17.** Images and quantitative analysis images for laser penetration ability verification under pork belly with different thicknesses (808 nm, 1.0 W/cm^2^, 4 min, IDG concentration 1.25 mg/mL). **Figure S18.** Images and quantitative analysis images for laser penetration ability verification under chicken breast with different thicknesses (808 nm, 1.0 W/cm^2^, 4 min, IDG concentration 1.25 mg/mL). **Figure S19.** Body temperature images and quantitative analysis of mice injected with different concentrations of IFDG under laser irradiation (808 nm, 1.0 W/cm^2^, 2 min). **Figure S20.** Body temperature images and quantitative analysis of mice injected with different concentrations of IDG under laser irradiation (808 nm, 1.0 W/cm^2^, 2 min). **Figure S21.** Quantitative analysis of cytotoxicity on different cell lines treated with different concentrations of IFDG, IDG, FA, and DOX (cell lines: HT-22, HEK293, IMR90m, and L929). **Figure S22.** After the mice were treated with IFDG, IDG, and normal saline, they were observed continuously for 14 days. Here is the statistics of whether the mice appeared angry, restless, coma, bulging eyes, or convulsions (BALB/c mice, n = 6). **Figure S23.** Quantitative analysis of aspartate aminotransferase (AST), creatine kinase (CK), lactate dehydrogenase (LDH), and albumin (ALB) of the mice in vivo after being injected with IFDG, IDG, and normal saline (BALB/c mice, n = 6). **Figure S24.** The MRI results of IFDG and IDG in the subcutaneous hepatic tumor model mice. MRI was performed at intervals, and the tumor area signals were quantitatively analyzed. The signal accumulation in the IFDG-treated group was significantly higher than that in the IDG-treated group. The signal accumulation reached the highest at 24 h, and the high signal could last for more than 48h, which was consistent with the case of orthotopic tumor mice (BALB/c-nu/nu mice, n = 3). **Figure S25.** The PAI results of IFDG and IDG in the subcutaneous hepatic tumor model mice. PAI was performed at intervals, and the tumor area signals were quantitatively analyzed. The signal accumulation in the IFDG-treated group was significantly higher than that in the IDG-treated group. The signal accumulation reached the highest at 24 h, and the high signal could last for more than 48h, which was consistent with the case of orthotopic tumor mice (BALB/c-nu/nu mice, n = 3). **Figure S26.** The NIR-II imaging results of IFDG and IDG in the subcutaneous hepatic tumor model mice. NIR-II imaging was performed at intervals, and the tumor area signals were quantitatively analyzed. The signal accumulation in the IFDG-treated group was significantly higher than that in the IDG-treated group. The signal accumulation reached the highest at 24 h, and the high signal could last for more than 48 h, which was consistent with the case of orthotopic tumor mice (BALB/c-nu/nu mice, n = 3). **Figure S27.** Images of organs (heart, liver, spleen, lung, kidney, and tumor) of mice with orthotopic hepatic tumors.**Figure S28.** Quantitative analysis of organs (heart, liver, spleen, lung, kidney, and tumor) of mice with orthotopic hepatic tumors. **Figure S29.** MRI images of mice with orthotopic hepatic tumors in the IFDG and IDG treated group during modeling, preoperative, and postoperative, respectively. **Figure S30.** Orthotopic liver cancer mice were injected with IFDG, IDG, and normal saline. PTT was then performed. Here are the tumor luciferase chemiluminescence imaging results after surgical resection of the mice tumors (n = 4). **Figure S31.** Images of the surgical resection process in mice with orthotopic hepatic tumors.

## Data Availability

All data generated and analyzed during this research are included in this published article.
