# Supplementary material for: Rational design of NIR-II molecule-engineered nanoplatform for preoperative downstaging and imaging-guided surgery of orthotopic hepatic tumor
Source: J Nanobiotechnology. 2023 Dec 18;21:489. doi: 10.1186/s12951-023-02263-w (PMC10726515; doi:10.1186/s12951-023-02263-w)
Supplement: Supplementary file 1 — Additional file 1: Figure S1. Schematic diagram of the synthetic route of IRFE-PEG-FA-DOTA-Gd. Figure S2. The hydrogen spectrum of compound 2. Figure S3. The carbon spectrum of compound 2. Figure S4. The hydrogen spectrum of compound 5. Figure S5. The carbon spectrum of compound 5. Figure S6. The hydrogen spectrum of compound 7. Figure S7. The carbon spectrum of compound 7. Figure S8. The hydrogen spectrum of compound 8. Figure S9. The carbon spectrum of compound 8. Figure S10. The hydrogen spectrum of compound 9. Figure S11. The hydrogen spectrum of compound 11 (IRFEP-FA-DOTA). Figure S12. The carbon spectrum of compound 11 (IRFEP-FA-DOTA). Figure S13. Gd3+ relaxation curves. Figure S14. Images and quantitative analysis images of IFDG in different concentrations under laser irradiation (808 nm, 1.0 W/cm2, 4 min). Figure S15. Images and quantitative analysis images of IDG in different concentrations under laser irradiation (808 nm, 1.0 W/cm2, 4 min). Figure S16. Verification laser penetration ability images and quantitative analysis images under different thicknesses of pork belly (808 nm, 1.0 W/cm2, 4 min, IFDG concentration 1.25 mg/mL). Figure S17. Images and quantitative analysis images for laser penetration ability verification under pork belly with different thicknesses (808 nm, 1.0 W/cm2, 4 min, IDG concentration 1.25 mg/mL). Figure S18. Images and quantitative analysis images for laser penetration ability verification under chicken breast with different thicknesses (808 nm, 1.0 W/cm2, 4 min, IDG concentration 1.25 mg/mL). Figure S19. Body temperature images and quantitative analysis of mice injected with different concentrations of IFDG under laser irradiation (808 nm, 1.0 W/cm2, 2 min). Figure S20. Body temperature images and quantitative analysis of mice injected with different concentrations of IDG under laser irradiation (808 nm, 1.0 W/cm2, 2 min). Figure S21. Quantitative analysis of cytotoxicity on different cell lines treated with different concentrati [file 12951_2023_2263_MOESM1_ESM.docx]

**Additional file 1**

**Rational design of NIR-II molecule-engineered nanoplatform for preoperative downstaging and imaging-guided surgery of orthotopic hepatic tumor**

Qi Pan^a,c^, Ke Li^d^, Xueqin Kang^e^, Kaixuan Li^c^, Zihe Cheng^d^, Yafei Wang^d^, Yuye Xu^d^, Lei Li^f^, Na Li^a^, Guilong Wu^a^, Sha Yang^a^, Shuo Qi^b^, Guodong Chen^b^, Xiaofeng Tan^a*^, Yonghua Zhan^e*^,Li Tang^g*^, Wenhua Zhan^h*^, and Qinglai Yang^a,b*^

^a^ Center for Molecular Imaging Probe, Hunan Province Key Laboratory of Cancer Cellular and Molecular Pathology, Cancer Research Institute, Hengyang Medical School, University of South China, Hengyang 421001, China.

^b^ Department of Hepatopancreatobiliary Surgery, The First Affiliated Hospital, Hengyang Medical School, University of South China, Hengyang, Hunan, 421001, China.

^c^ Medical Imaging Department, The Second Affiliated Hospital of Xi'an Medical University, Xi'an 710038, China.

^d^ Xi'an Key Laboratory for Prevention and Treatment of Common Aging Diseases, Translational and Research Centre for Prevention and Therapy of Chronic Disease, Institute of Basic and Translational Medicine, Xi'an Medical University, Xi'an, 710021, China.

^e^ Engineering Research Center of Molecular & Neuro Imaging of the Ministry of Education, School of Life Science and Technology, Xidian University, Xi’an 710126, China.

^f^ Radiology Department, Ninth Affiliated Hospital of Medical College of Xi’an Jiaotong University, Xi’an 710054, China.

^g^ Key Laboratory of Tropical Medicinal Plant Chemistry of Ministry of Education, College of Chemistry and Chemical Engineering, Hainan Normal University, Haikou 571158, China.

^h^ Department of Radiation Oncology, General Hospital of Ningxia Medical University, Yinchuan 750004, China

* Corresponding authors.

E-mail addresses: tanxiaofeng@usc.edu.cn (Xiaofeng Tan), yhzhan@xidian.edu.cn (Yonghua Zhan), tanglicq@outlook.com (Li Tang), zhanwhgood@163.com (Wenhua Zhan), qingyu513@126.com (Qinglai Yang).

**Additional figures**

**Figure S1.** Schematic diagram of the synthetic route of IRFE-PEG-FA-DOTA-Gd.


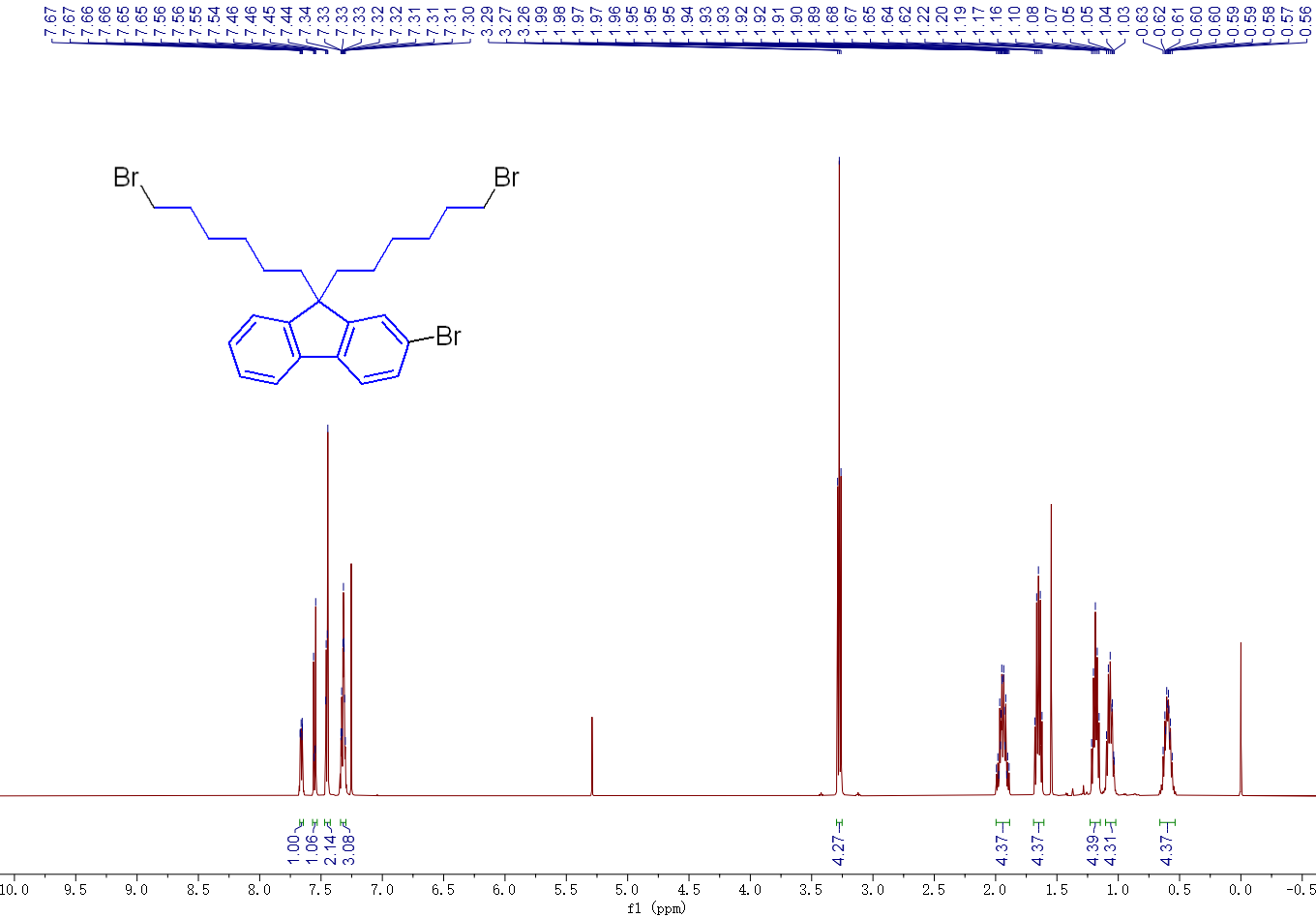


**Figure S2**. The hydrogen spectrum of compound 2.


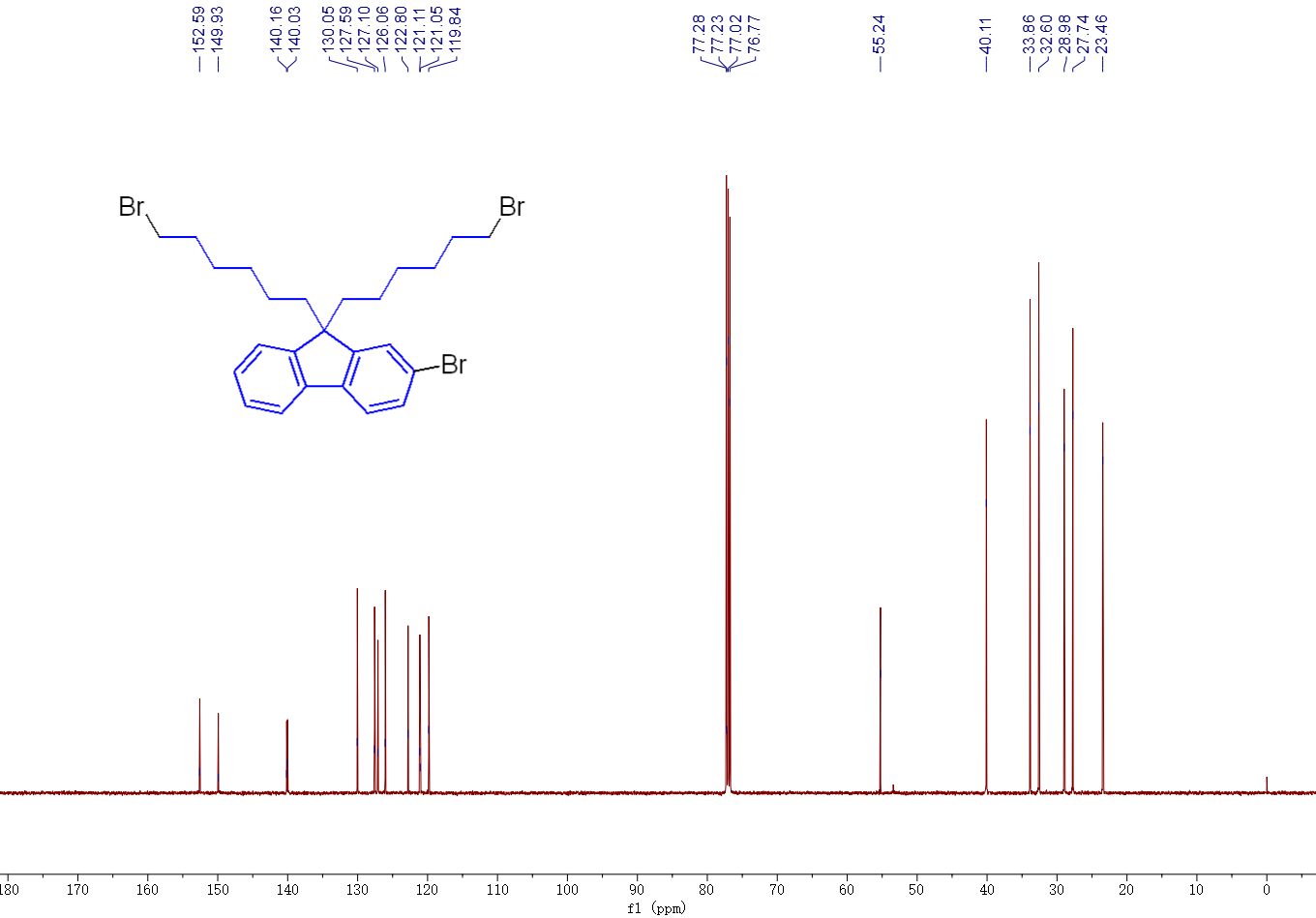


**Figure S3**. The carbon spectrum of compound 2.


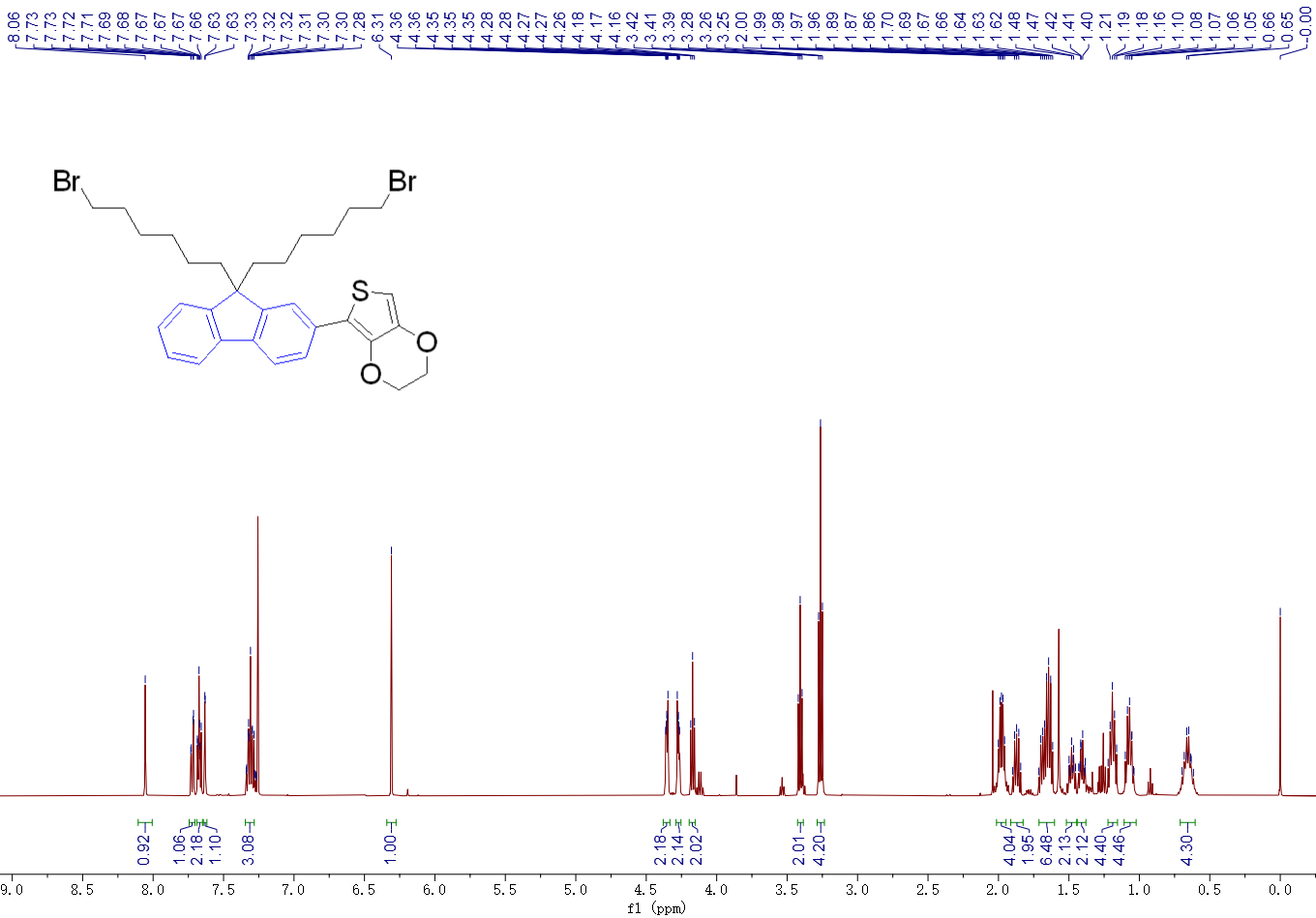


**Figure S4**. The hydrogen spectrum of compound 5.


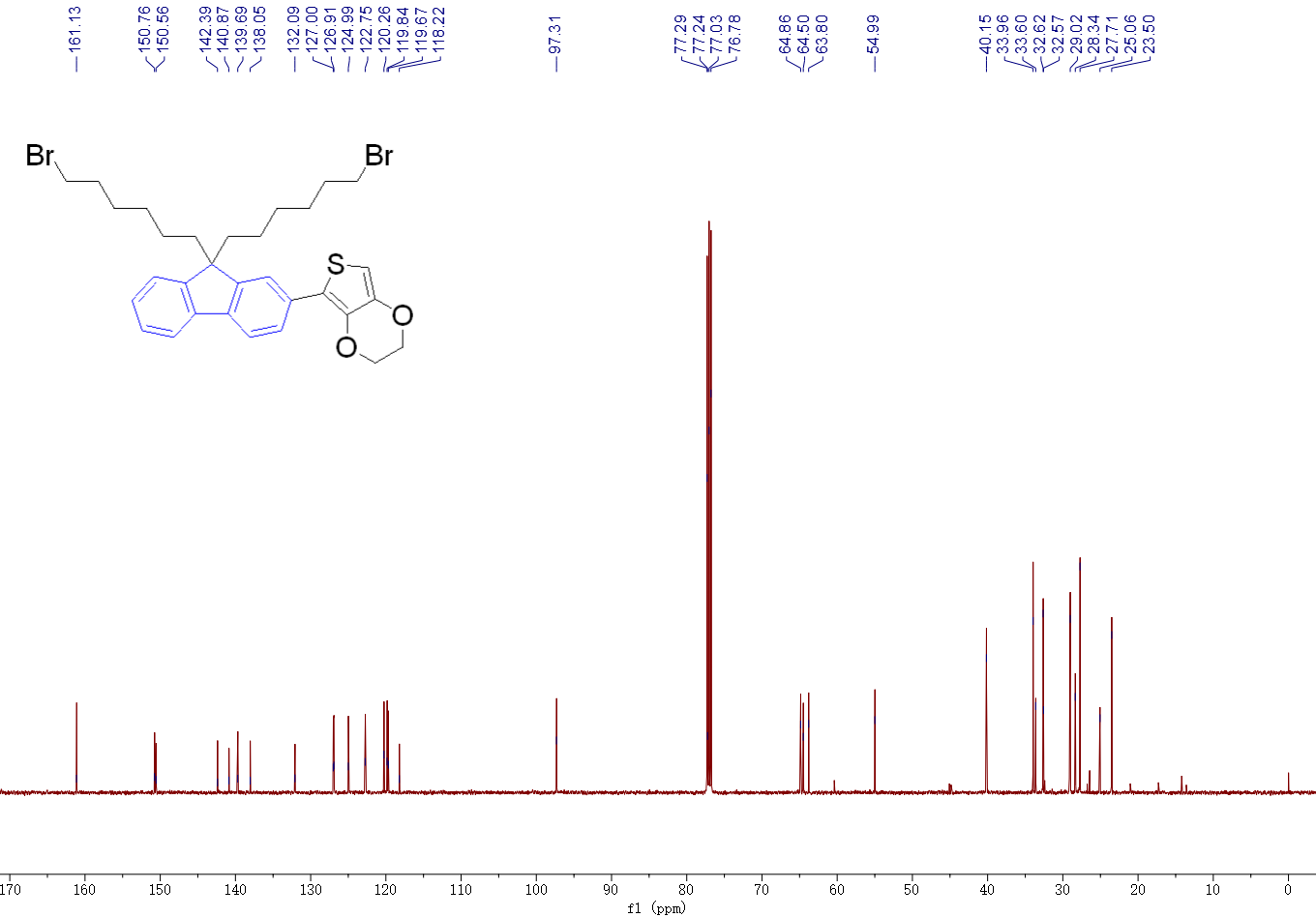


**Figure S5**. The carbon spectrum of compound 5.


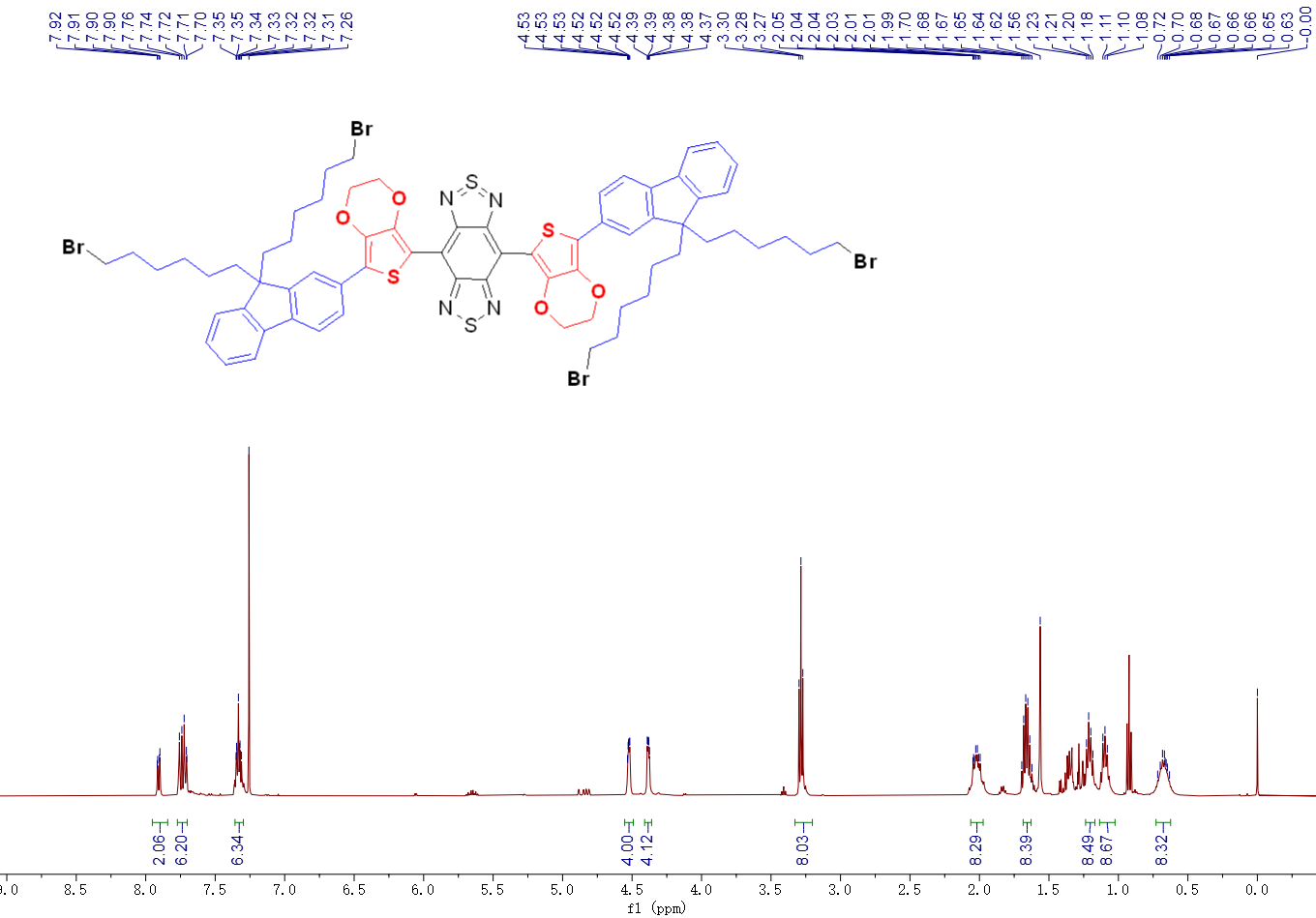


**Figure S6**. The hydrogen spectrum of compound 7.

**Figure S7**. The carbon spectrum of compound 7.

**Figure S8**. The hydrogen spectrum of compound 8.

**Figure S9**. The carbon spectrum of compound 8.

**Figure S10**. The hydrogen spectrum of compound 9.

**Figure S11**. The hydrogen spectrum of compound 11 (IRFEP-FA-DOTA).

**Figure S12**. The carbon spectrum of compound 11 (IRFEP-FA-DOTA).

**Figure S13**. Gd^3+^ relaxation curves.


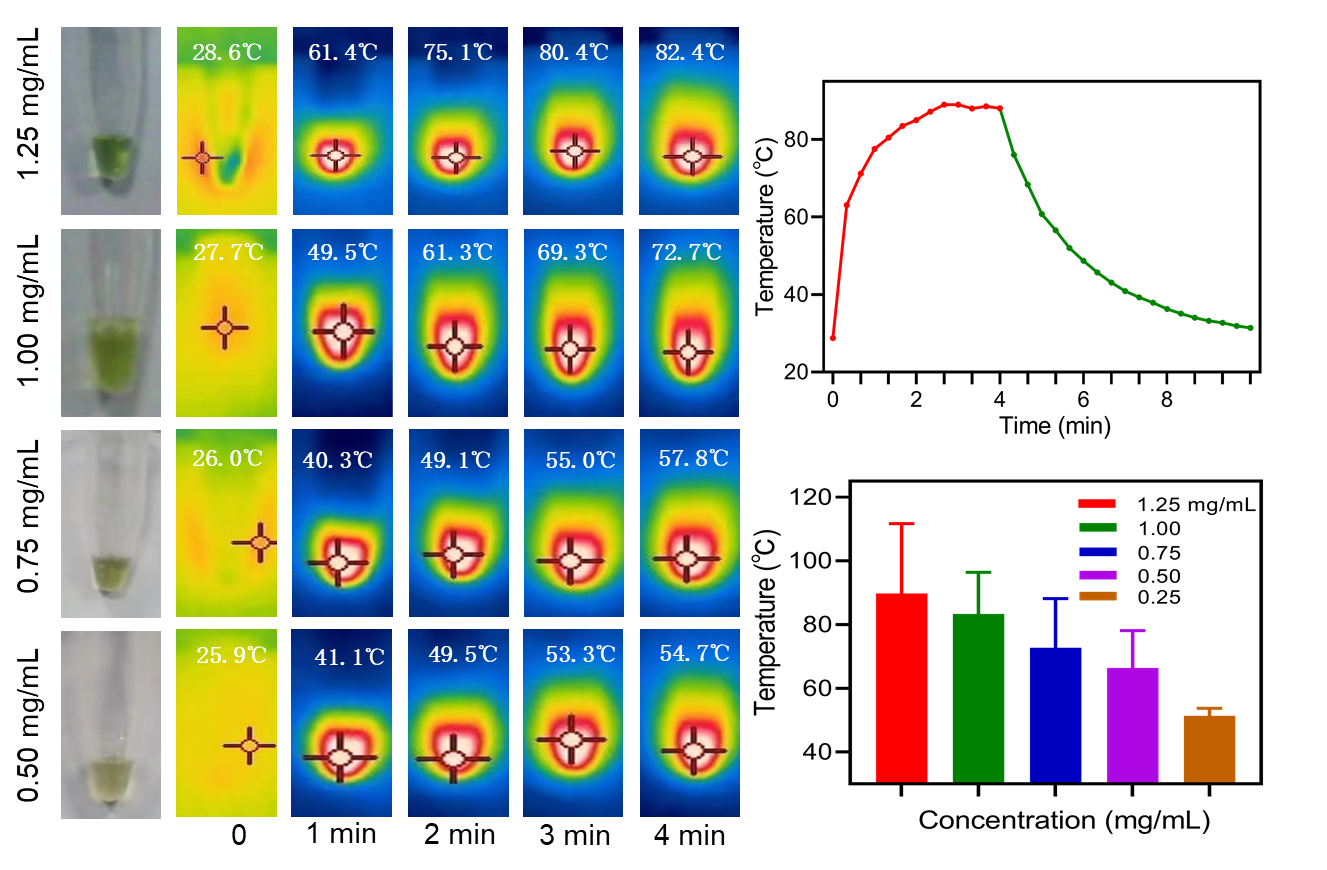


**Figure S14**. Images and quantitative analysis images of IFDG in different concentrations under laser irradiation (808 nm, 1.0 W/cm^2^, 4 min).


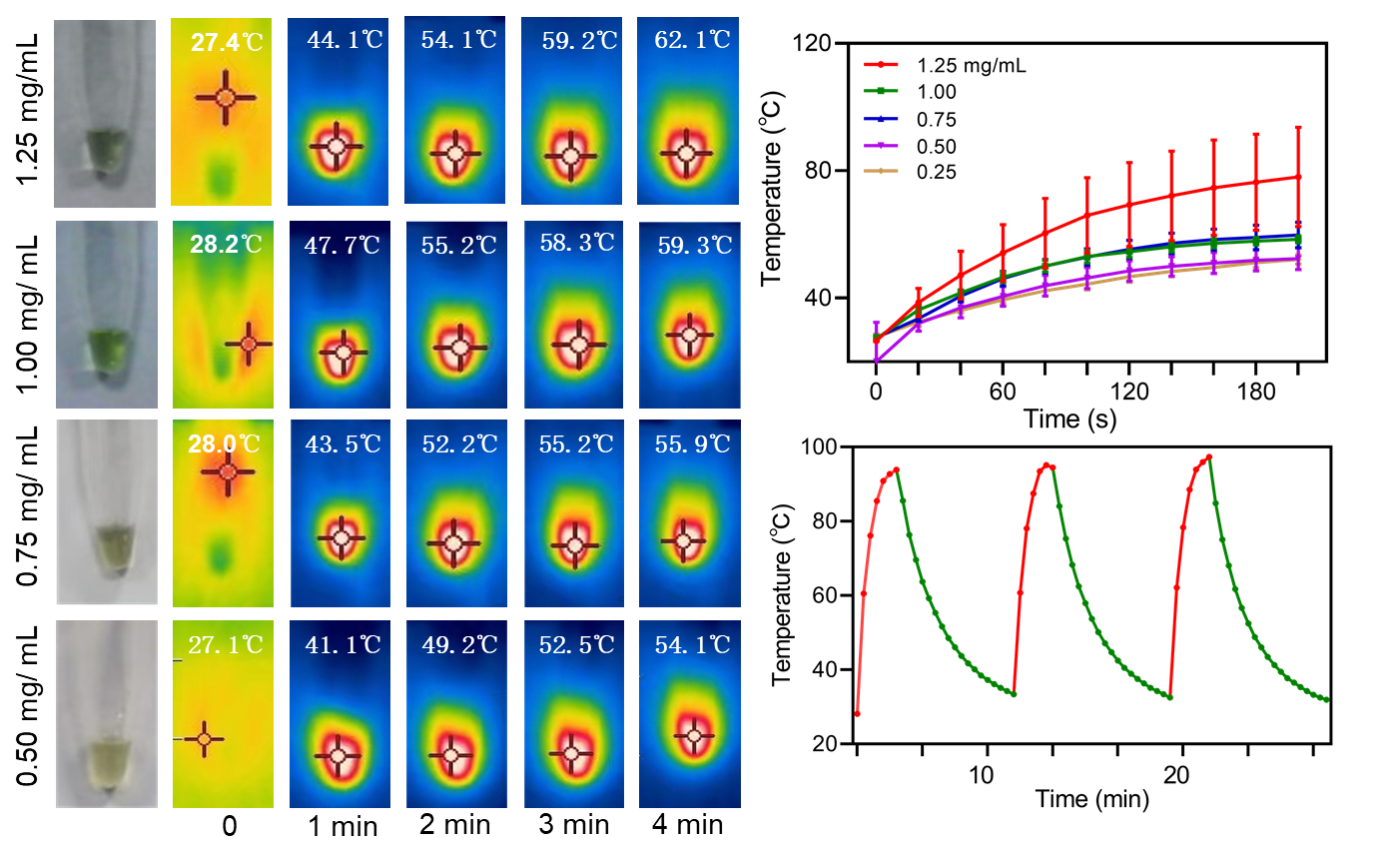


**Figure S15**. Images and quantitative analysis images of IDG in different concentrations under laser irradiation (808 nm, 1.0 W/cm^2^, 4 min).


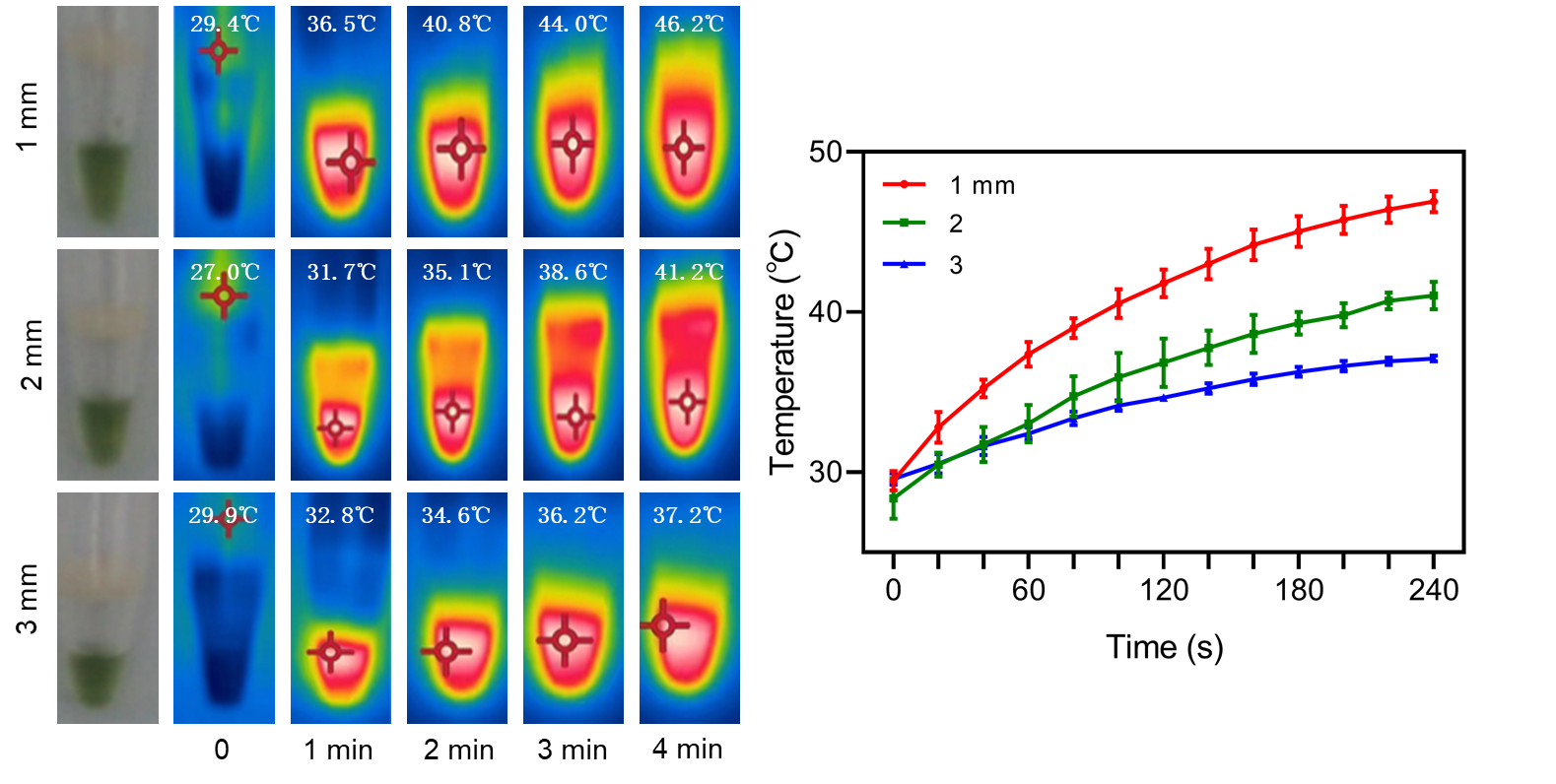


**Figure S16**. Verification laser penetration ability images and quantitative analysis images under different thicknesses of pork belly (808 nm, 1.0 W/cm^2^, 4 min, IFDG concentration 1.25 mg/mL).


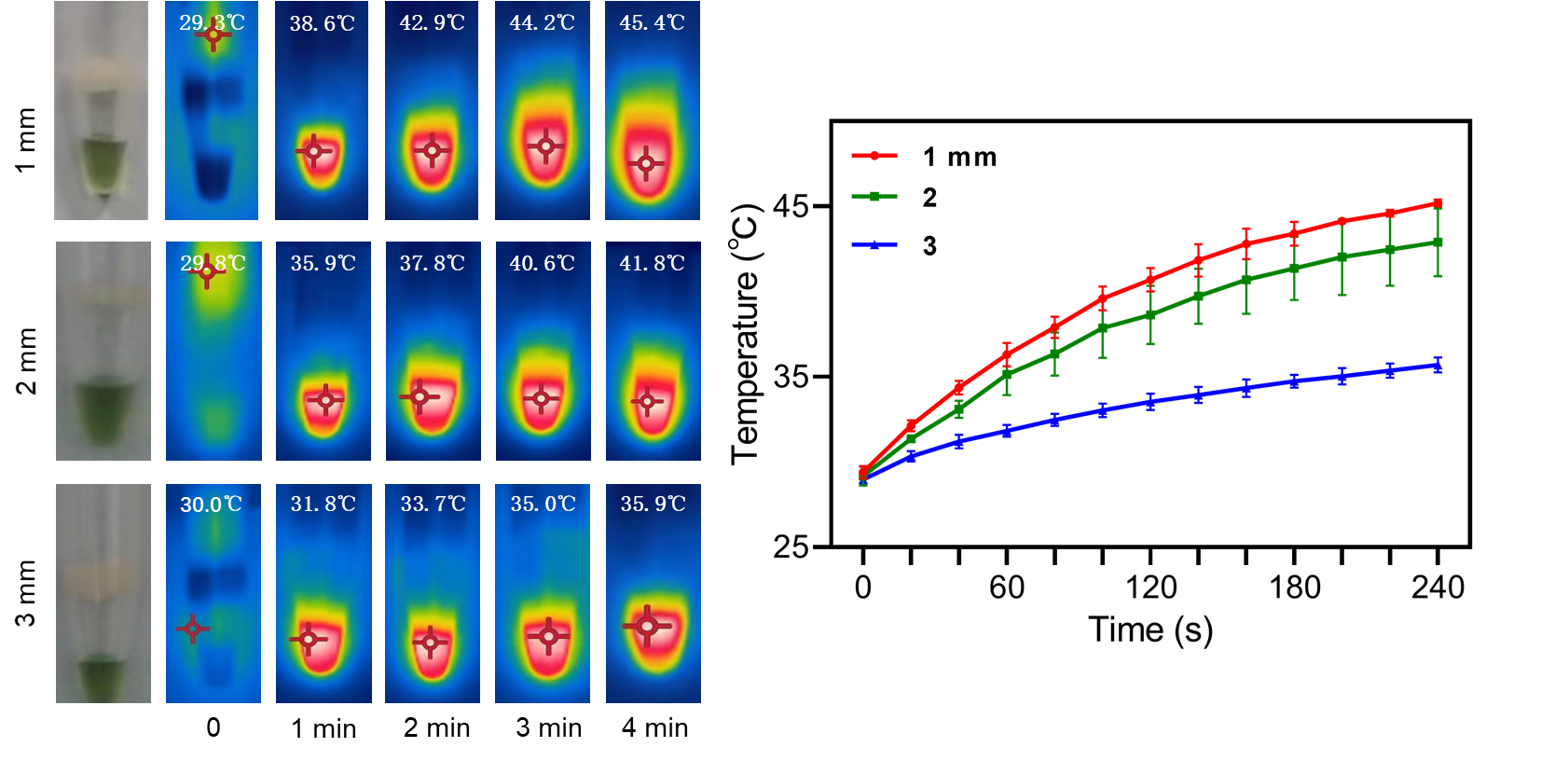


**Figure S17**. Images and quantitative analysis images for laser penetration ability verification under pork belly with different thicknesses (808 nm, 1.0 W/cm^2^, 4 min, IDG concentration

1.25 mg/mL).


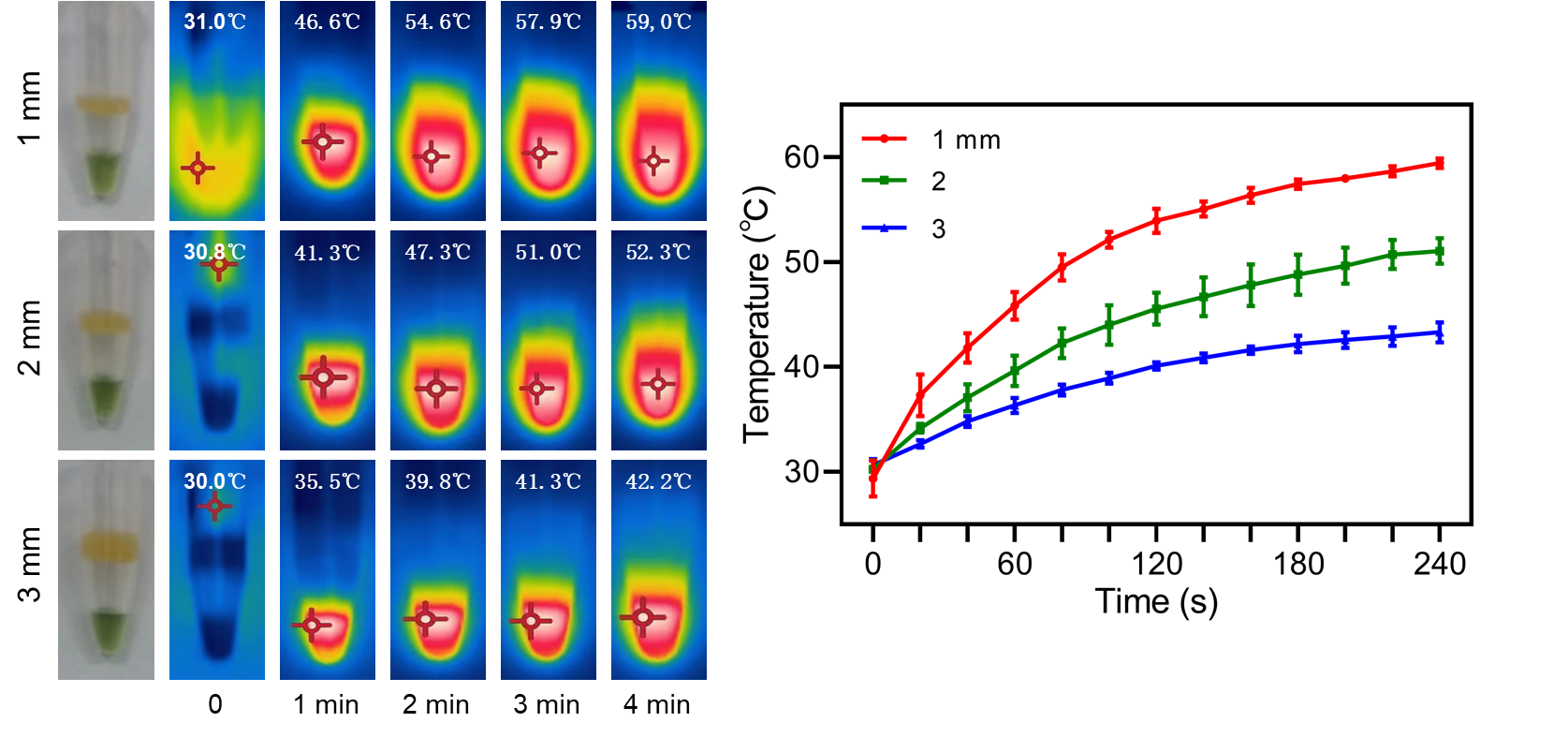


**Figure S18**. Images and quantitative analysis images for laser penetration ability verification under chicken breast with different thicknesses (808 nm, 1.0 W/cm^2^, 4 min, IDG concentration 1.25 mg/mL).


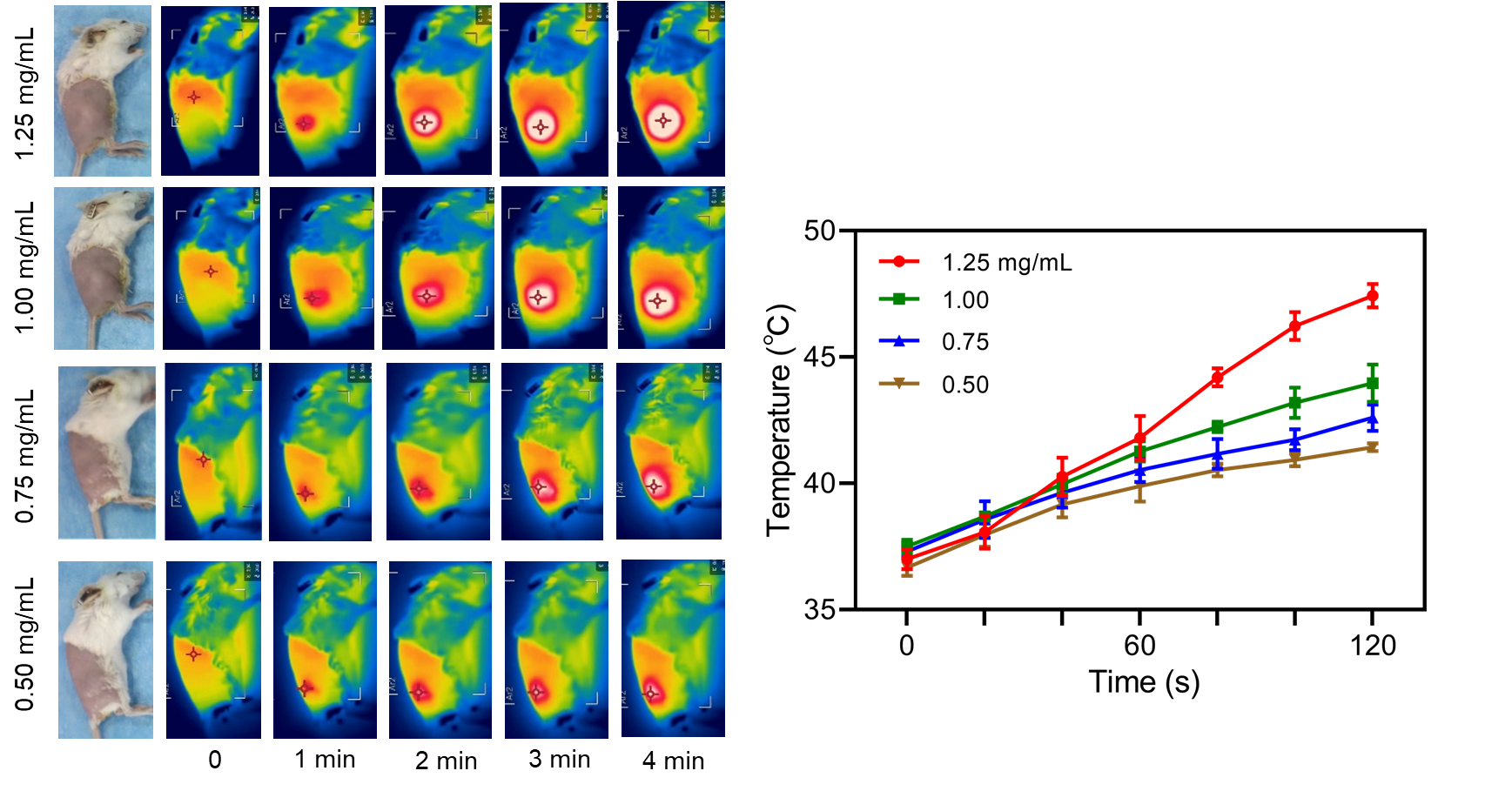


**Figure S19**. Body temperature images and quantitative analysis of mice injected with different concentrations of IFDG under laser irradiation (808 nm, 1.0 W/cm^2^, 2 min).


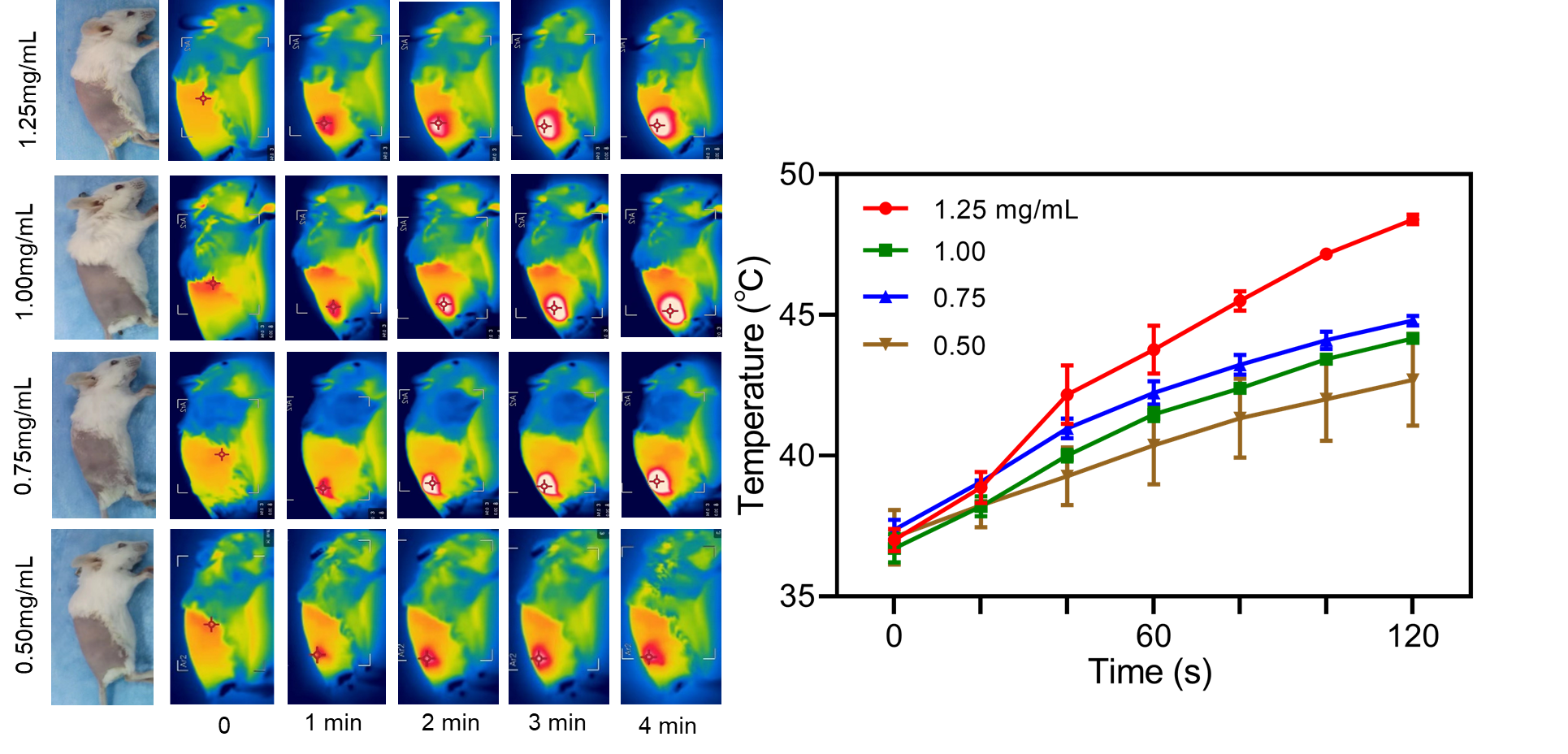
 **Figure S20**. Body temperature images and quantitative analysis of mice injected with different concentrations of IDG under laser irradiation (808 nm, 1.0 W/cm^2^, 2 min).


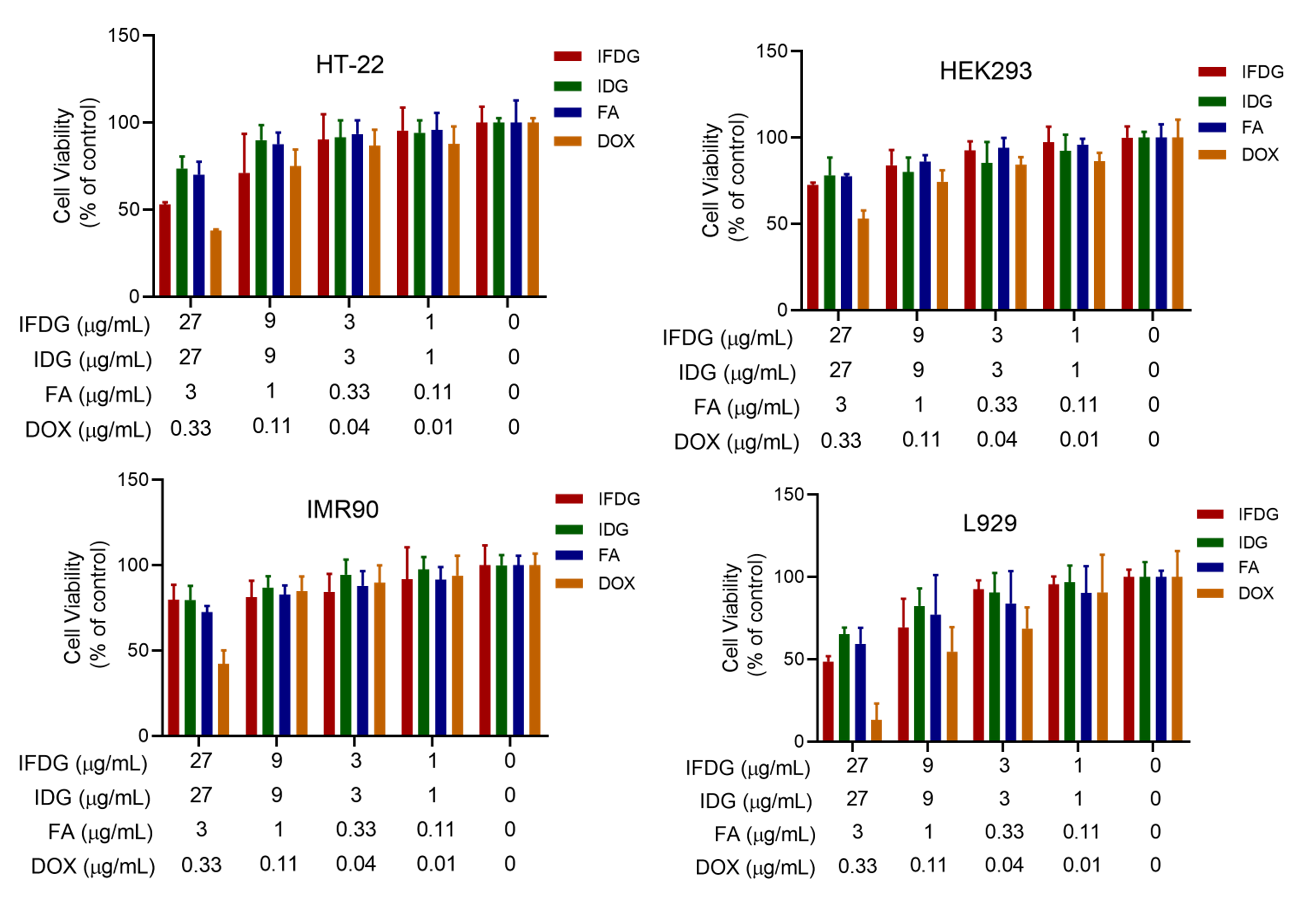


**Figure S21**. Quantitative analysis of cytotoxicity on different cell lines treated with different concentrations of IFDG, IDG, FA, and DOX (cell lines: HT-22, HEK293, IMR90m, and L929).


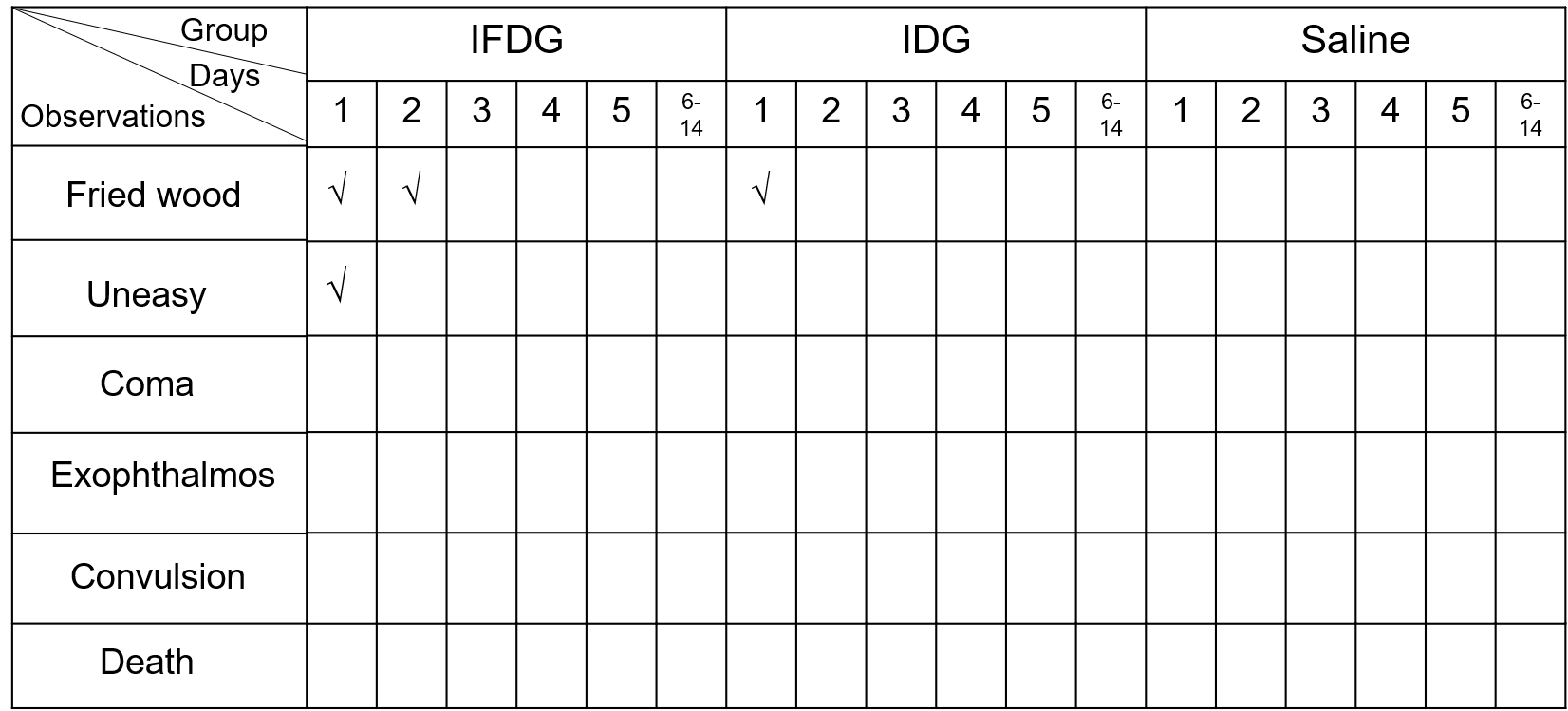


**Figure S22**. After the mice were treated with IFDG, IDG, and normal saline, they were observed continuously for 14 days. Here is the statistics of whether the mice appeared angry, restless, coma, bulging eyes, or convulsions (BALB/c mice, n=6).


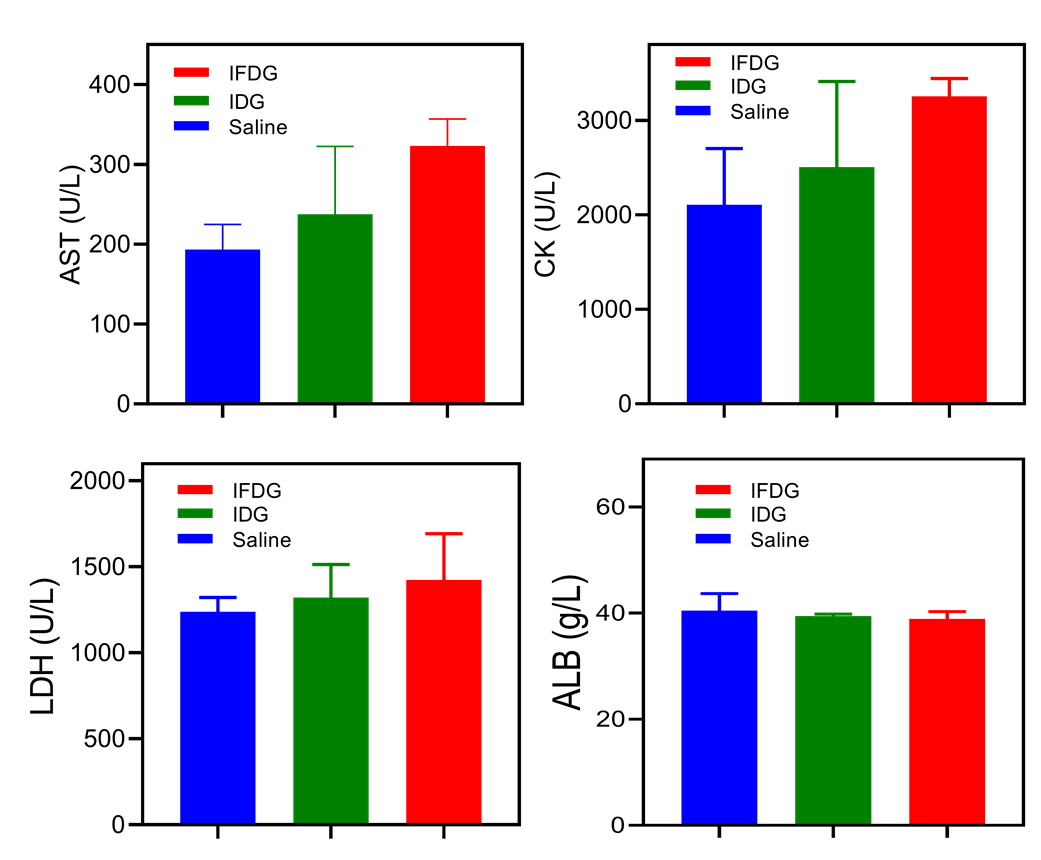


**Figure S23**. Quantitative analysis of aspartate aminotransferase (AST), creatine kinase (CK), lactate dehydrogenase (LDH), and albumin (ALB) of the mice in vivo after being injected with IFDG, IDG, and normal saline (BALB/c mice, n=6).


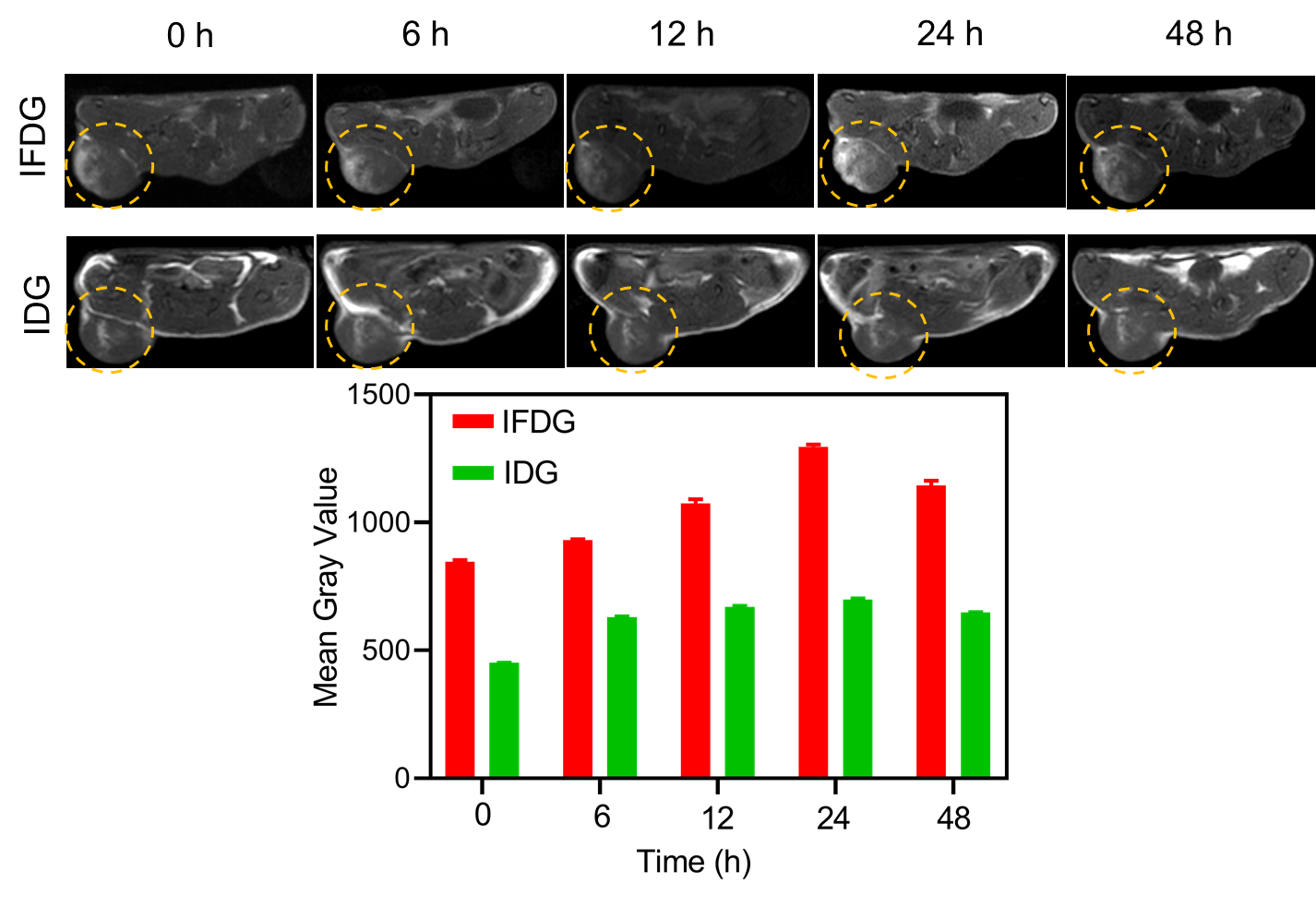


**Figure S24**. The MRI results of IFDG and IDG in the subcutaneous hepatic tumor model mice. MRI was performed at intervals, and the tumor area signals were quantitatively analyzed. The signal accumulation in the IFDG-treated group was significantly higher than that in the IDG-treated group. The signal accumulation reached the highest at 24 h, and the high signal could last for more than 48h, which was consistent with the case of orthotopic tumor mice (BALB/c-nu/nu mice, n=3).


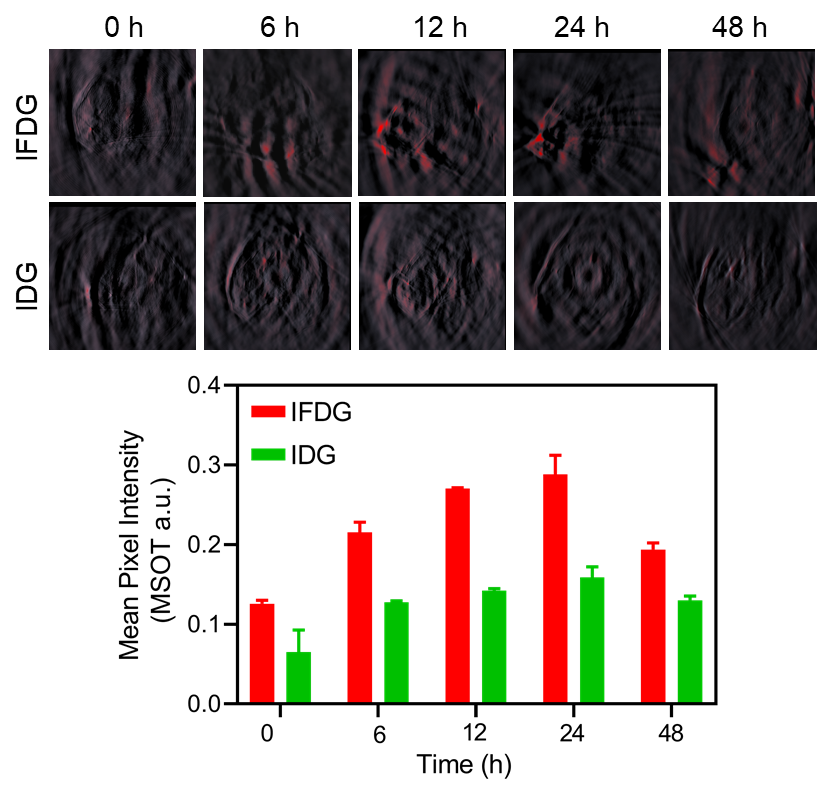


**Figure S25.** The PAI results of IFDG and IDG in the subcutaneous hepatic tumor model mice. PAI was performed at intervals, and the tumor area signals were quantitatively analyzed. The signal accumulation in the IFDG-treated group was significantly higher than that in the IDG-treated group. The signal accumulation reached the highest at 24 h, and the high signal could last for more than 48h, which was consistent with the case of orthotopic tumor mice (BALB/c-nu/nu mice, n=3).


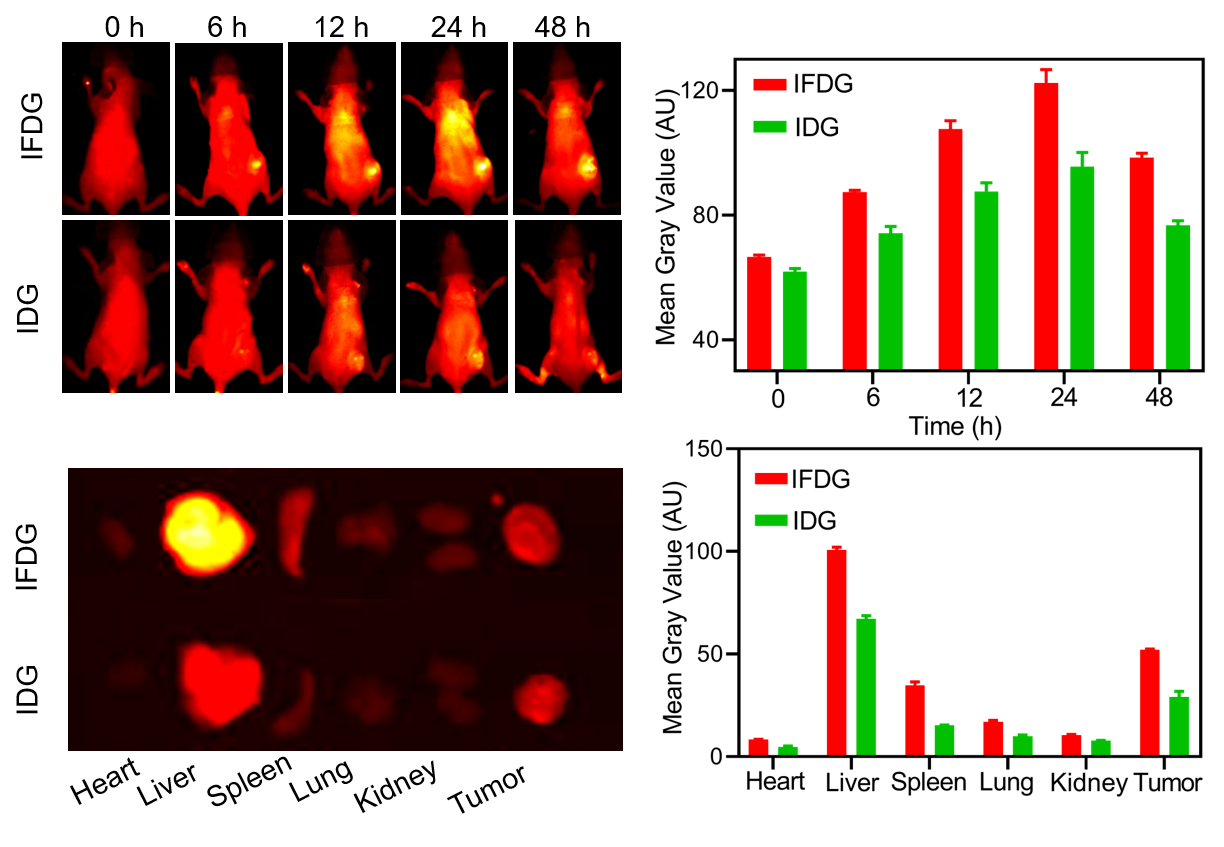


**Figure S26**. The NIR-II imaging results of IFDG and IDG in the subcutaneous hepatic tumor model mice. NIR-II imaging was performed at intervals, and the tumor area signals were quantitatively analyzed. The signal accumulation in the IFDG-treated group was significantly higher than that in the IDG-treated group. The signal accumulation reached the highest at 24 h, and the high signal could last for more than 48 h, which was consistent with the case of orthotopic tumor mice (BALB/c-nu/nu mice, n=3).


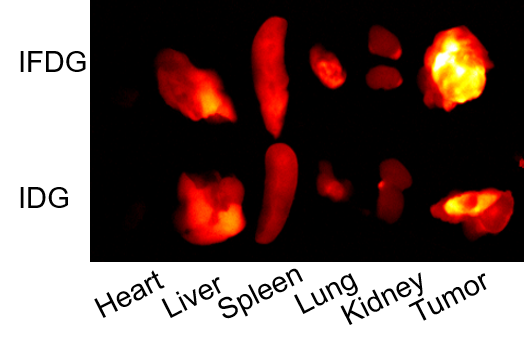


**Figure S27.** Images of organs (heart, liver, spleen, lung, kidney, and tumor) of mice with orthotopic hepatic tumors.


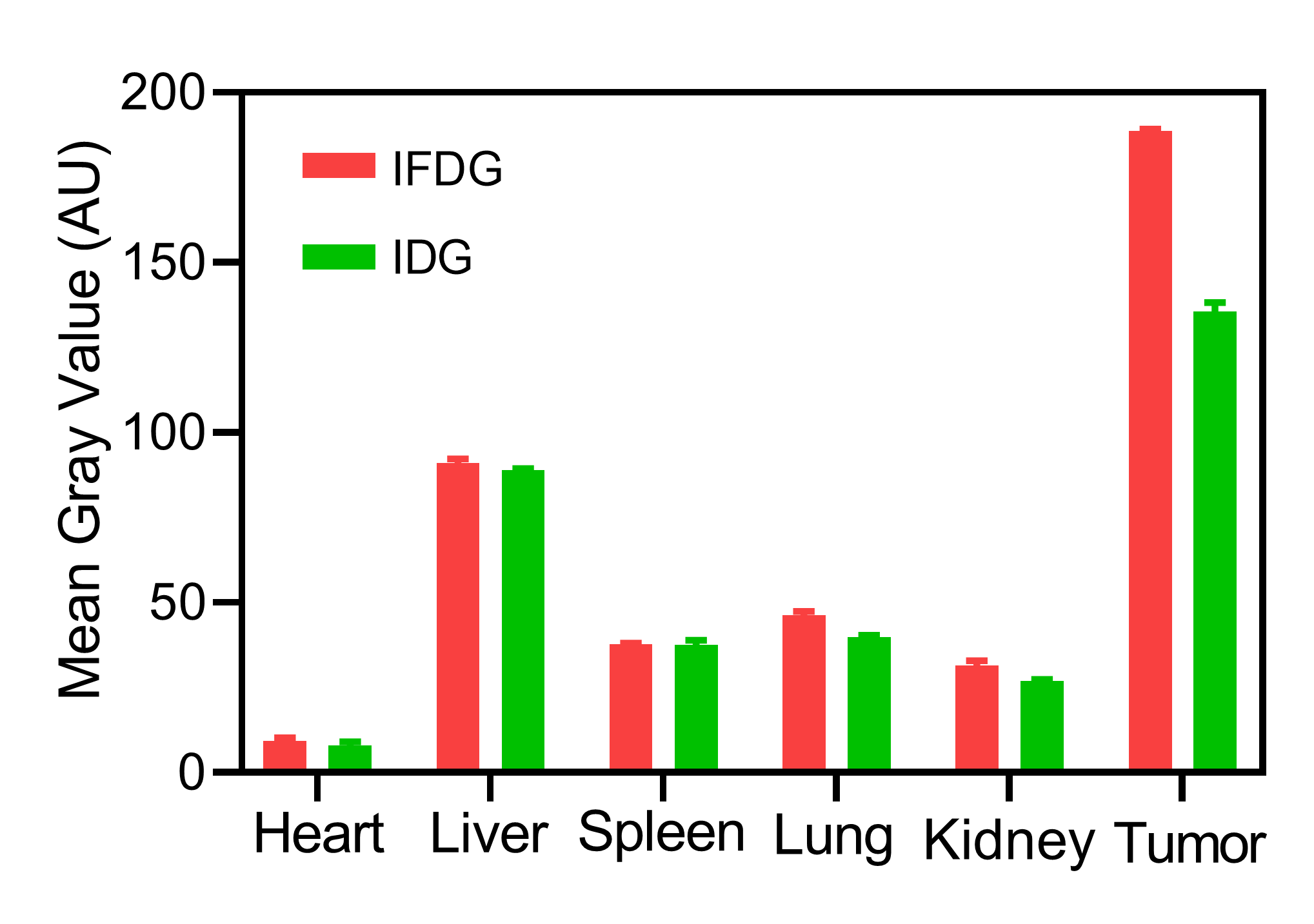


**Figure S28**. Quantitative analysis of organs (heart, liver, spleen, lung, kidney, and tumor) of mice with orthotopic hepatic tumors.


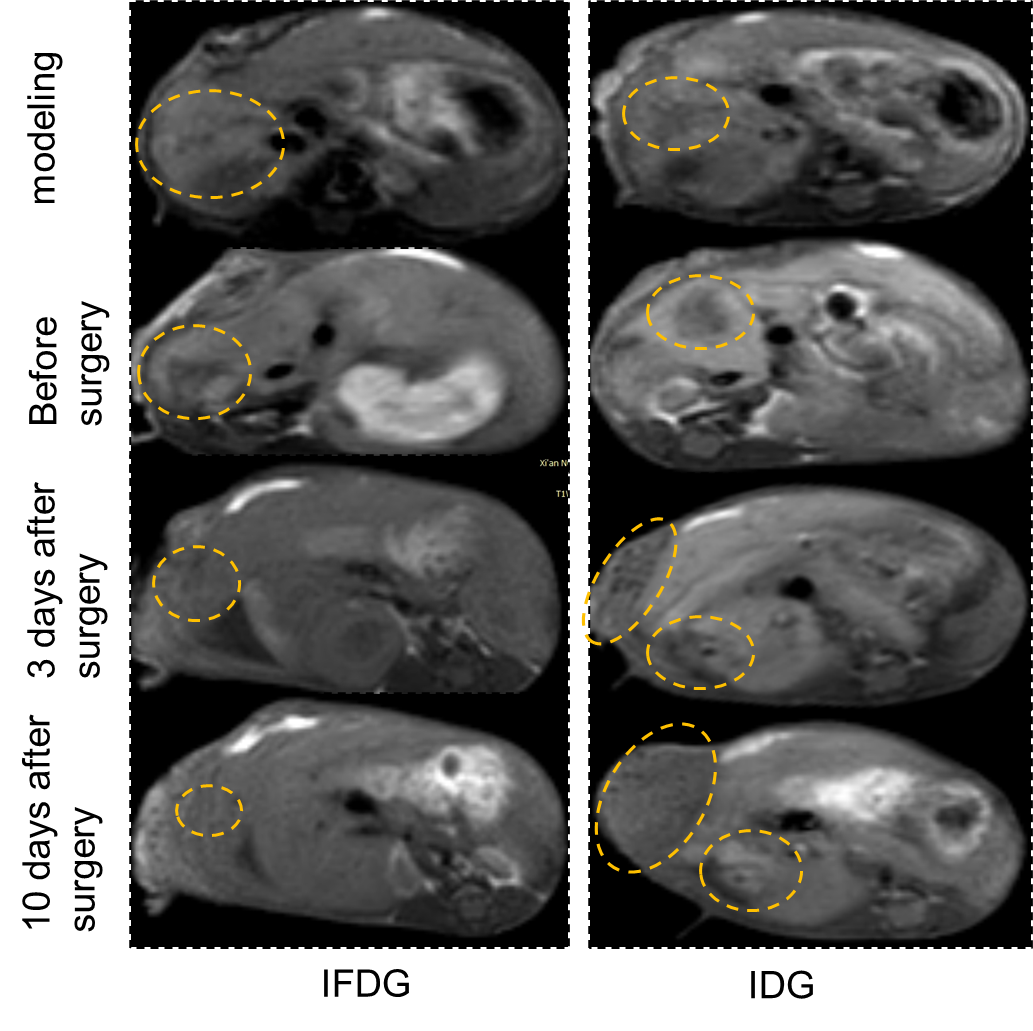


**Figure S29**. MRI images of mice with orthotopic hepatic tumors in the IFDG and IDG treated group during modeling, preoperative, and postoperative, respectively.


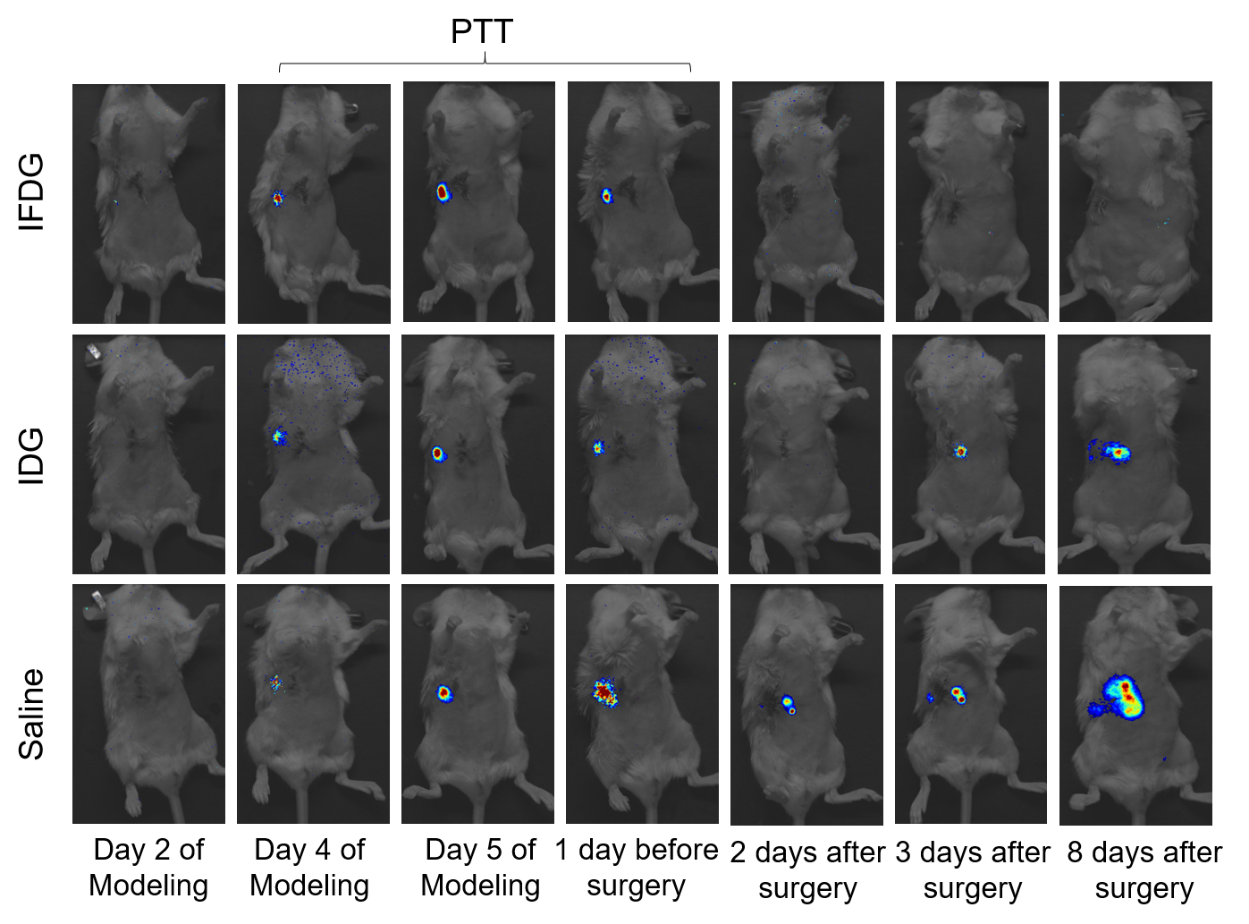


**Figure S30**. Orthotopic liver cancer mice were injected with IFDG, IDG, and normal saline. PTT was then performed. Here are the tumor luciferase chemiluminescence imaging results after surgical resection of the mice tumors (n=4).


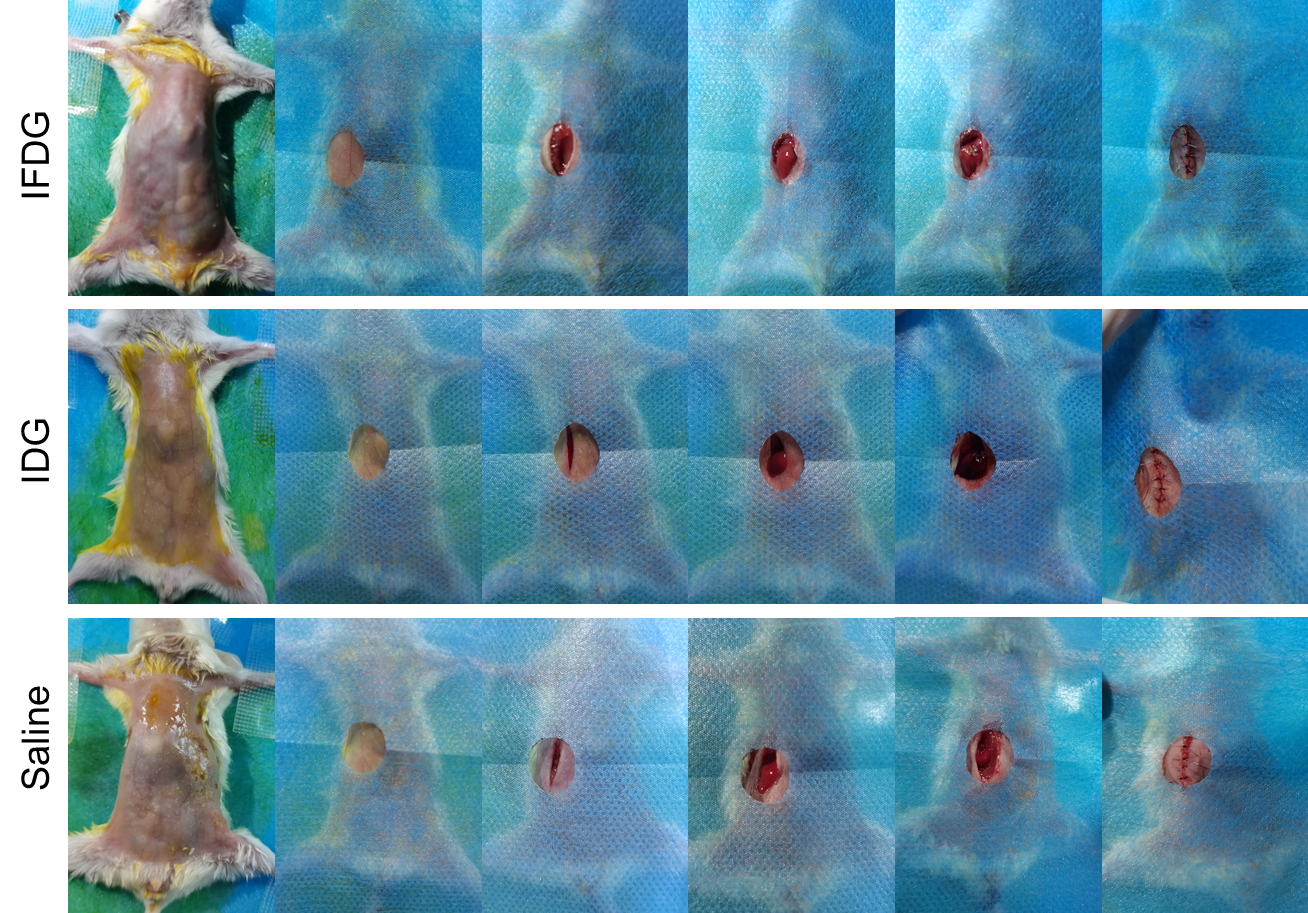


**Figure S31**. Images of the surgical resection process in mice with orthotopic hepatic tumors.
